# Identification of Critical Amino Acid Residues Required for the Polar Localization of a Rice Manganese Transporter

**DOI:** 10.1111/pbi.70708

**Published:** 2026-06-25

**Authors:** Noriyuki Konishi, Jian Feng Ma

**Affiliations:** ^1^ Institute of Plant Science and Resources Okayama University Kurashiki Japan

**Keywords:** amino acid residue, clathrin‐mediated endocytosis, Mn, OsNramp5, polar localization, rice, root, transporter

## Abstract

Rice has developed an efficient system for manganese (Mn) uptake, mediated by two distinct transporters, OsNramp5 and OsMTP9. These transporters exhibit polar localization at the root exodermis and endodermis; however, the mechanisms underlying their polar localization and their role in Mn uptake remain unclear. Here, we identified key amino acid residues critical for the polar localization of OsNramp5 at the distal side. Through analysis of chimeric proteins between OsNramp5 and its non‐polar homologues, we found that the C‐terminal cytosolic region of OsNramp5 is essential for its polar localization. Site‐directed mutagenesis further revealed that aspartate 500 and four valine residues at positions 494, 495, 498 and 506 are crucial for polarity. Substitution of these valine residues with isoleucine, leucine, phenylalanine, or threonine partially or fully maintained polar localization, whereas substitution with alanine, serine, or asparagine resulted in loss of polarity. These findings suggest that β‐branching and high hydrophobicity of amino acid side chains are likely required for OsNramp5 polarity. Furthermore, we found that adaptor protein 2‐dependent clathrin‐mediated endocytosis is not involved in the polar localization of OsNramp5. Finally, we provided experimental evidence showing the significant role of OsNramp5 polarity in efficient Mn uptake in rice; plants expressing non‐polarly localized OsNramp5 exhibited reduced Mn uptake compared to those with polarly localized OsNramp5. In addition, we found that cadmium accumulation in shoots could be reduced by manipulating OsNramp5 polarity in combination with its overexpression, without a growth penalty.

## 1 Introduction

The uptake of mineral nutrients essential for plant growth and development is mediated by various transporters expressed in roots. To facilitate the directional and radial transport of minerals from the soil solution to the stele and subsequently to the shoots, a coordinated transport system is required (Barberon and Geldner 2014). Recent studies have shown that this system often involves both influx and efflux transporters that exhibit polar localization (Ma and Yamaji 2015; Shao et al. 2017; Yoshinari and Takano 2017; Robe and Barberon 2023). For example, in Arabidopsis, the boron (B) transporters AtNIP5;1 and AtBOR1 localize to the distal and proximal sides of epidermal and endodermal cells, respectively (Takano et al. 2010). Similarly, in rice, transporters for silicon (Si) (OsLsi1 and OsLsi2), B (OsLsi1 and OsBOR1), and manganese (Mn) (OsNramp5 and OsMTP9) exhibit polar localization at the distal and proximal sides of both the exodermis and endodermis (Ma et al. 2006, 2007; Sasaki et al. 2012; Ueno et al. 2015; Huang, Konishi, et al. 2022). These influx‐efflux transporter pairs establish a radial pathway for the uptake of mineral elements. Additional examples of polarly localized mineral transporters include the nitrate transporter AtNRT2.4 (Kiba et al. 2012), iron transporters AtIRT1 (Barberon et al. 2014) and AtZIP8 (Robe et al. 2025), zinc transporter AtZIP3 (Robe et al. 2025), ammonium transporter OsAMT1s (Konishi and Ma 2021), phosphate transporters StPT2 (Gordon‐Weeks et al. 2003) and GmPT4 (Guo et al. 2022), and the ferric‐mugineic acid complex transporter ZmYS1 (Ueno et al. 2009), although their efflux counterparts have not been identified.

The mechanisms underlying polarity of mineral transporters have been investigated in both Arabidopsis and rice. In Arabidopsis, clathrin‐mediated endocytosis (CME) is required for the polar localization of AtNIP5;1, AtBOR1 and AtIRT1 (Alassimone et al. 2010; Yoshinari et al. 2016, 2019; Wang et al. 2017; Spielmann et al. 2025). Inhibition of CME via chemical treatment, dominant‐negative suppression of dynamin‐related proteins, or knockout of adaptor protein 2 (AP2) complex subunits disrupted the polarity of AtNIP5;1 and AtBOR1 in root epidermal cells (Alassimone et al. 2010; Yoshinari et al. 2016, 2019; Wang et al. 2017). Similarly, AP2‐dependent constitutive CME is required for AtIRT1 polarity (Spielmann et al. 2025). The AP2 complex directly binds to the C‐terminal cytosolic region of AtBOR1 (Yoshinari et al. 2019) and to YxxΦ motifs in the cytosolic loop of AtIRT1 (Spielmann et al. 2025). Phosphorylation of the TPG repeat in the N‐terminus of AtNIP5;1 is necessary for its polar localization via constitutive endocytosis (Wang et al. 2017). Additionally, CME of AtNIP5;1—but not AtBOR1—requires myosin XI, a motor protein involved in cytoplasmic streaming (Liu et al. 2025). The FYVE domain‐containing protein FYVE1 has also been implicated in AtIRT1 polarity by binding to its cytosolic loop (Barberon et al. 2014).

In contrast, studies on rice transporters OsLsi1, OsLsi2 and OsBOR1 suggest that CME is not involved in their polar localization (Huang, Konishi, et al. 2022; Konishi et al. 2022). Neither dominant‐negative suppression of dynamin‐related proteins nor knockout of the AP2 mu subunit affected the polarity of these transporters, indicating distinct mechanisms in rice from Arabidopsis (Huang, Konishi, et al. 2022; Konishi et al. 2022). Further work revealed that OsLsi1 polarity requires Ile18, Ile285, and positively charged residues in its N‐ and C‐terminal cytosolic regions (Konishi et al. 2023). However, mechanisms governing the polarity of other mineral transporters in rice remain largely unknown.

In this study, we investigated the molecular basis of OsNramp5 polar localization in rice. OsNramp5, a member of the NATURAL RESISTANCE‐ASSOCIATED MACROPHAGE PROTEIN (Nramp) family, is the primary Mn uptake transporter and also transports other divalent metals such as cadmium (Cd), cobalt (Co), lead (Pb), and zinc (Zn) (Sasaki et al. 2012; Ishikawa et al. 2012; Chang et al. 2022; Huang et al. 2025; Huang et al. 2026). It localizes to the distal side of both the exodermis and endodermis in roots (Sasaki et al. 2012). Knockout of *OsNramp5* severely reduces Mn uptake and impairs plant growth, particularly under Mn‐limited conditions (Sasaki et al. 2012). Using chimeric protein analysis and site‐directed mutagenesis, we identified amino acid residues critical for OsNramp5 trafficking and polar localization. We also provide evidence that polar localization is essential for efficient Mn uptake in rice.

## 2 Results

### 2.1 AP2‐Dependent CME Is Not Required for the Polar Localization of OsNramp5

To investigate whether AP2‐dependent CME is involved in the polar localization of OsNramp5, we used a knockout mutant of the *OsAP2M* gene, which encodes one of the subunits of the AP2 heterotetrameric complex (Konishi et al. 2022; Huang, Konishi, et al. 2022). Immunostaining with OsNramp5 antibody showed that, similar to the WT, OsNramp5 was polarly localized at the distal side of the exodermis and endodermis of roots in *ap2m* mutants (Figure S1a–d). This result indicates that the AP2‐dependent CME was not required for the polar localization of OsNramp5.

### 2.2 Ectopic Expression of *OsNramp5* Homologues in Root Exodermis and Endodermis

**FIGURE 1:**
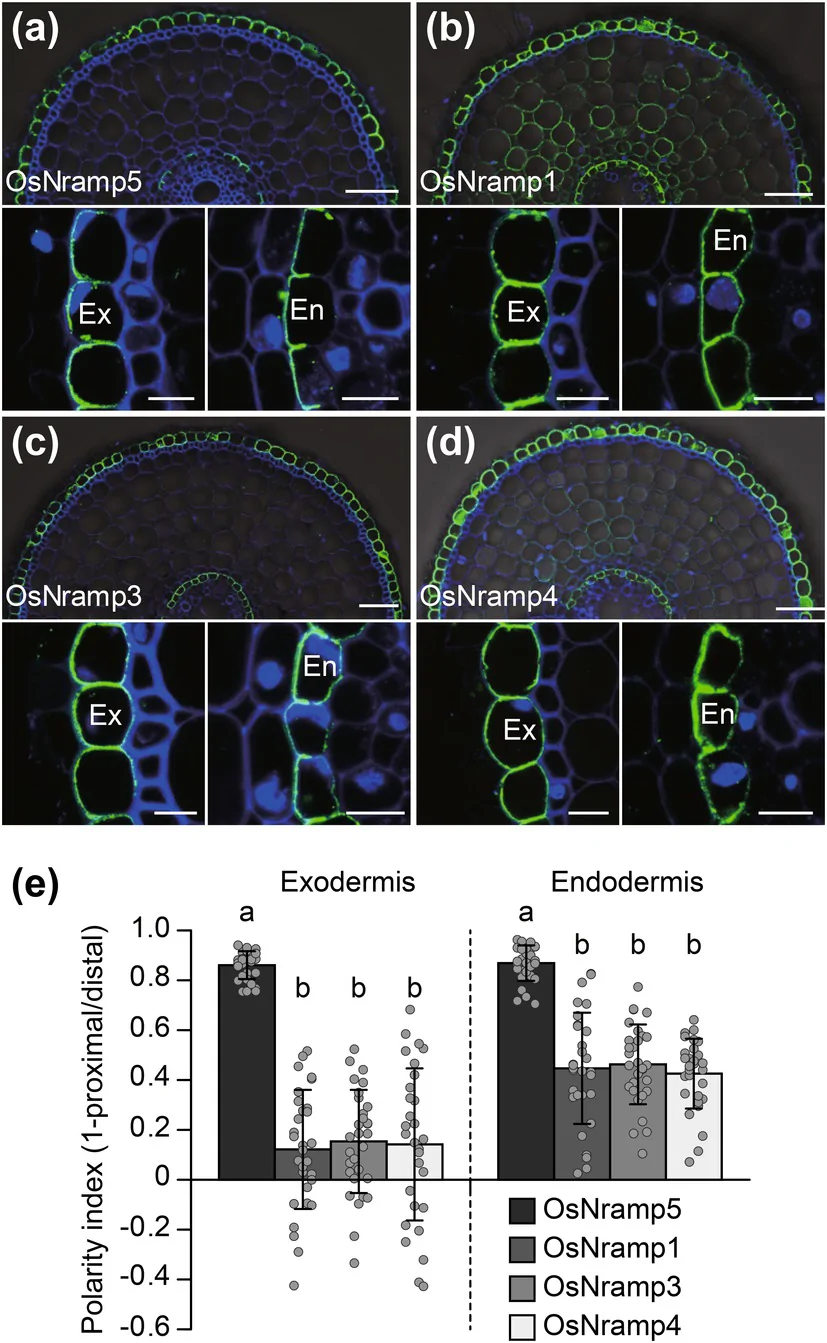
Cellular localization of OsNramp transporter members ectopically expressed under the control of the *OsLsi1* promoter. (a–d) Localization of OsNramp5‐Flag (a), OsNramp1‐Flag (b), OsNramp3‐Flag (c), and OsNramp4‐Flag (d) in the root exodermis and endodermis. Cross‐sections of the mature region (15–20 mm from the root tip) of crown roots were used for immunostaining with an anti‐Flag antibody. Enlarged images of the exodermis (Ex) and endodermis (En) in (a–d) are shown below at each image (a–d). Green signals represent transporter localization; blue signals indicate cell wall autofluorescence and DAPI‐stained nuclei. Scale bars: 50 μm (whole root) and 10 μm (enlarged). (e) Polarity index of the OsNramp members shown in (a–d) in the exodermis and endodermis. Data are from 30 cells per sample from ≥ 5 root slices. Different letters denote significant differences (*p* < 0.05, Tukey–Kramer test).

The rice genome contains seven Nramp family members (Thomine and Schroeder 2004). Among these, only OsNramp5, which is expressed in the root exodermis and endodermis, exhibits polar localization (Sasaki et al. 2012). In contrast, other members—including OsNramp1, OsNramp3 and OsNramp4—are primarily expressed in other tissues and do not show polarity (Xia et al. 2010; Yamaji et al. 2013; Chang, Huang, Konishi, et al. 2020; Chang, Huang, Yamaji, et al. 2020). To determine whether polar localization results from tissue‐specific expression, we ectopically expressed C‐terminally Flag‐tagged versions of *OsNramp5*, *OsNramp1*, *OsNramp3* and *OsNramp4* in a *nramp5* loss‐of‐function mutant (Ishikawa et al. 2012) under the control of the *OsLsi1* promoter, which drives specific expression in the root exodermis and endodermis (Ma et al. 2006; Huang, Konishi, et al. 2022; Huang et al. 2024). Immunostaining with an anti‐Flag antibody revealed that OsNramp5 was clearly localized to the distal side, whereas OsNramp1, OsNramp3, and OsNramp4 did not show polar localization, despite being detected in both exodermal and endodermal cells (Figure 1a–d).

To quantify the polarity of these OsNramp proteins, we calculated a polarity index as previously described (Konishi et al. 2023). The polarity index of OsNramp5 was close to one in both the exodermis and endodermis, indicating that the signal was predominantly detected on the distal side (Figure 1e). In contrast, the polarity indices for OsNramp1, OsNramp3 and OsNramp4 were near zero in the exodermis and approximately 0.5 in the endodermis (Figure 1e), indicating a lack of polarity or only weak polarity in these cell types, respectively. These findings suggest that polar localization is not conferred by tissue‐specific expression but rather by intrinsic features of the transporter. Based on these results, we generated chimeric proteins between OsNramp5 and its non‐polar homologues to narrow the region required for polar localization.

### 2.3 N‐Terminal Region of OsNramp5 Is Not Required for Its Polar Localization

**FIGURE 2:**
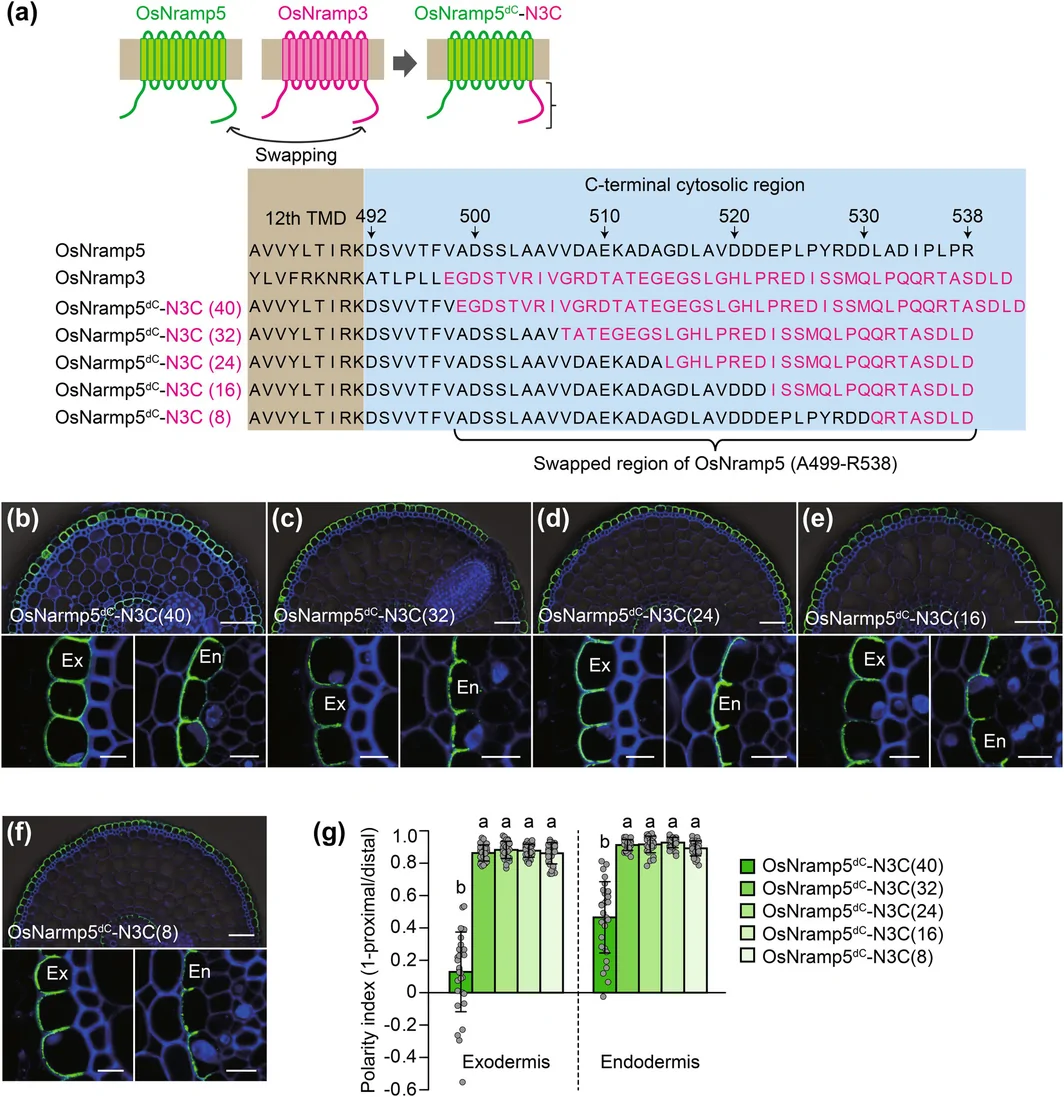
Cellular localization of chimeric proteins generated by swapping C‐terminal regions between OsNramp5 and OsNramp3. (a) Schematic representation of chimeric protein design. Green indicates OsNramp5 topology; magenta indicates OsNramp3 topology. Magenta sequences represent regions swapped from OsNramp3. TMD, transmembrane domain. (b–f) Localization of chimeric proteins: OsNramp5^dC^‐N3C (40) (b), OsNramp5^dC^‐N3C (32) (c), OsNramp5^dC^‐N3C (24) (d), OsNramp5^dC^‐N3C (16) (e), and OsNramp5^dC^‐N3C (8) (f). Cross‐sections of mature crown roots from transgenic plants were immunostained. Green signals represent chimeric proteins; blue signals indicate cell wall autofluorescence and DAPI‐stained nuclei. Enlarged images of exodermis (Ex) and endodermis (En) are shown below at each image (b–f). Scale bars: 50 μm (whole root) and 10 μm (enlarged). (g) Polarity index of chimeric proteins in exodermis and endodermis. Data are from 30 cells per sample from ≥ 5 root slices. Different letters denote significant differences (*p* < 0.05, Tukey–Kramer test).

OsNramp5 is predicted to contain 12 transmembrane domains and large cytosolic regions at both the N‐ and C‐termini (Figure 2a). We hypothesized that these cytosolic domains might contribute to polar localization. To test whether the N‐terminal cytosolic region is involved, we replaced the N‐terminus of OsNramp5 with that of OsNramp1 (N1N‐OsNramp5^dN^), OsNramp3 (N3N‐OsNramp5^dN^), or OsNramp4 (N4N‐OsNramp5^dN^), which differ in sequence (Figure S2a). Chimeric proteins carrying the N‐terminus of OsNramp1 or OsNramp4 localized to the plasma membrane and maintained polar localization at the distal side of both exodermis and endodermis (Figure S2b,d–f). However, attachment of the OsNramp3 N‐terminus resulted in endomembrane accumulation (Figure S2c). Conversely, fusing the N‐terminus of OsNramp5 to an N‐terminally deleted OsNramp3 (N5N‐OsNramp3^dN^) did not confer polar localization (Figure S2e,f). These results indicate that the N‐terminal region of OsNramp5 is not required for its polar localization.

### 2.4 C‐Terminal Cytosolic Region Is Required for Polar Localization of OsNramp5

We next examined the role of the C‐terminal cytosolic region. We swapped various segments of the OsNramp5 C‐terminus with corresponding regions from OsNramp3 (Figure 2a). Swapping the last 40 amino acids (Ala499‐Arg538) resulted in a significant reduction in the polarity index in both exodermis and endodermis, whereas shorter swaps (Val507‐Arg538) had little effect (Figure 2b–g). These findings suggest that the region between Ala499 and Val506 is necessary for polar localization.

**FIGURE 3:**
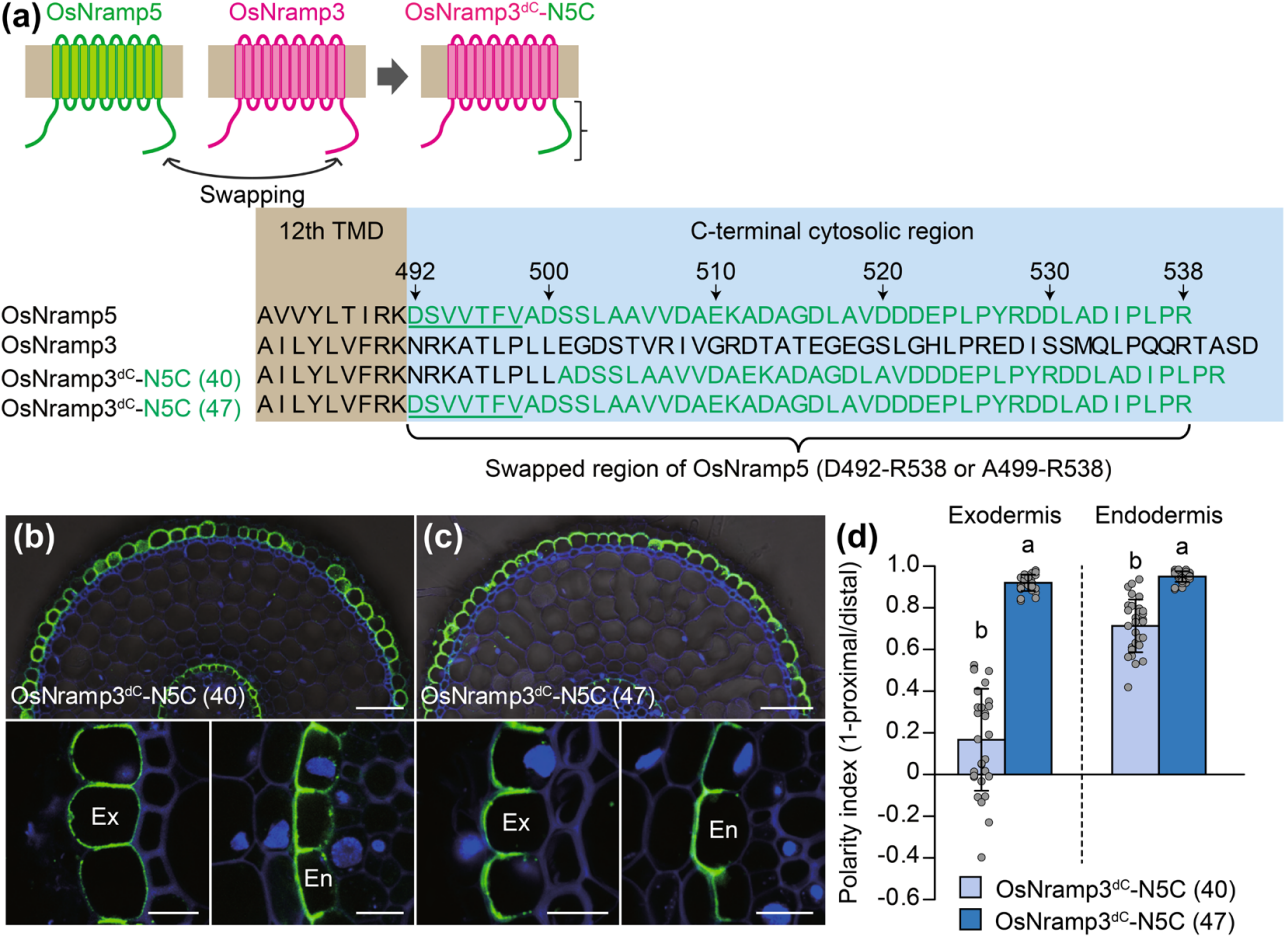
Localization of chimeric proteins generated by swapping C‐terminal regions between OsNramp3 and OsNramp5. (a) Schematic representation of chimeric protein design. Green indicates OsNramp5 topology; magenta indicates OsNramp3 topology. Green sequences represent regions swapped from OsNramp5. Underlined green sequences indicate a different attachment region. TMD, transmembrane domain. (b, c) Localization of OsNramp3^dC^‐N5C (40) (b) and OsNramp3^dC^‐N5C (47) (c) in roots of transgenic lines. Cross‐sections of mature crown roots were immunostained. Green signals represent chimeric proteins; blue signals indicate cell wall autofluorescence and DAPI‐stained nuclei. Enlarged images of exodermis (Ex) and endodermis (En) are shown below at each image (b, c). Scale bars: 50 μm (whole root) and 10 μm (enlarged). (d) Polarity index of chimeric proteins in exodermis and endodermis. Data are from 30 cells per sample from ≥ 5 root slices. Different letters indicate significant differences (*p* < 0.05, Tukey–Kramer test).

To test whether the C‐terminal region of OsNramp5 is sufficient to confer polarity to a non‐polar homologue, we attached the OsNramp5 C‐terminus (Ala499‐Arg538) to a C‐terminally truncated OsNramp3 (Figure 3a). Although this region is necessary for polarity (Figure 2), it was not sufficient to polarize OsNramp3 (Figure 3b,d). However, when the entire C‐terminal region (Asp492‐Arg538) was attached, the resulting chimeric protein exhibited polar localization (Figure 3c,d), indicating that the full C‐terminal region is sufficient to confer polarity. Combined with the OsNramp5‐ and OsNramp3‐based swaps, these results suggest that the region between Asp492 and Val506 at the C‐terminus is critical for OsNramp5 polarity.

### 2.5 Multiple Residues in the C‐Terminus Are Involved in Polar Localization

To identify specific residues required for polarity, we performed Ala‐scanning mutagenesis across the Asp492‐Val506 region (Figure S3a). Substitutions in the regions Asp492‐Val506, Asp492‐Asp500 and Asp492‐Val495 led to endomembrane accumulation (Figure S3b–d). Notably, a single substitution of Asp492 (D492A) also caused endomembrane retention (Figure S3e), and its signal colocalized with the ER marker HDEL (Figure S4), indicating that Asp492 is essential for ER‐to‐plasma membrane trafficking. This residue may be involved in quality control or COPII‐mediated export (Marti et al. 2010; Strasser 2018). This residue was then preserved in subsequent polarity analyses.

When residues in the Val494‐Val506 region were substituted with Ala, OsNramp5 lost polar localization, whereas substitutions in Phe497‐Val506, Asp500‐Val506 and Thr496‐Val498 resulted in partial polarity loss specifically in the exodermis (Figure 3f–j). These observations suggest that multiple residues between Val494 and Val506 contribute to polarity.

**FIGURE 4:**
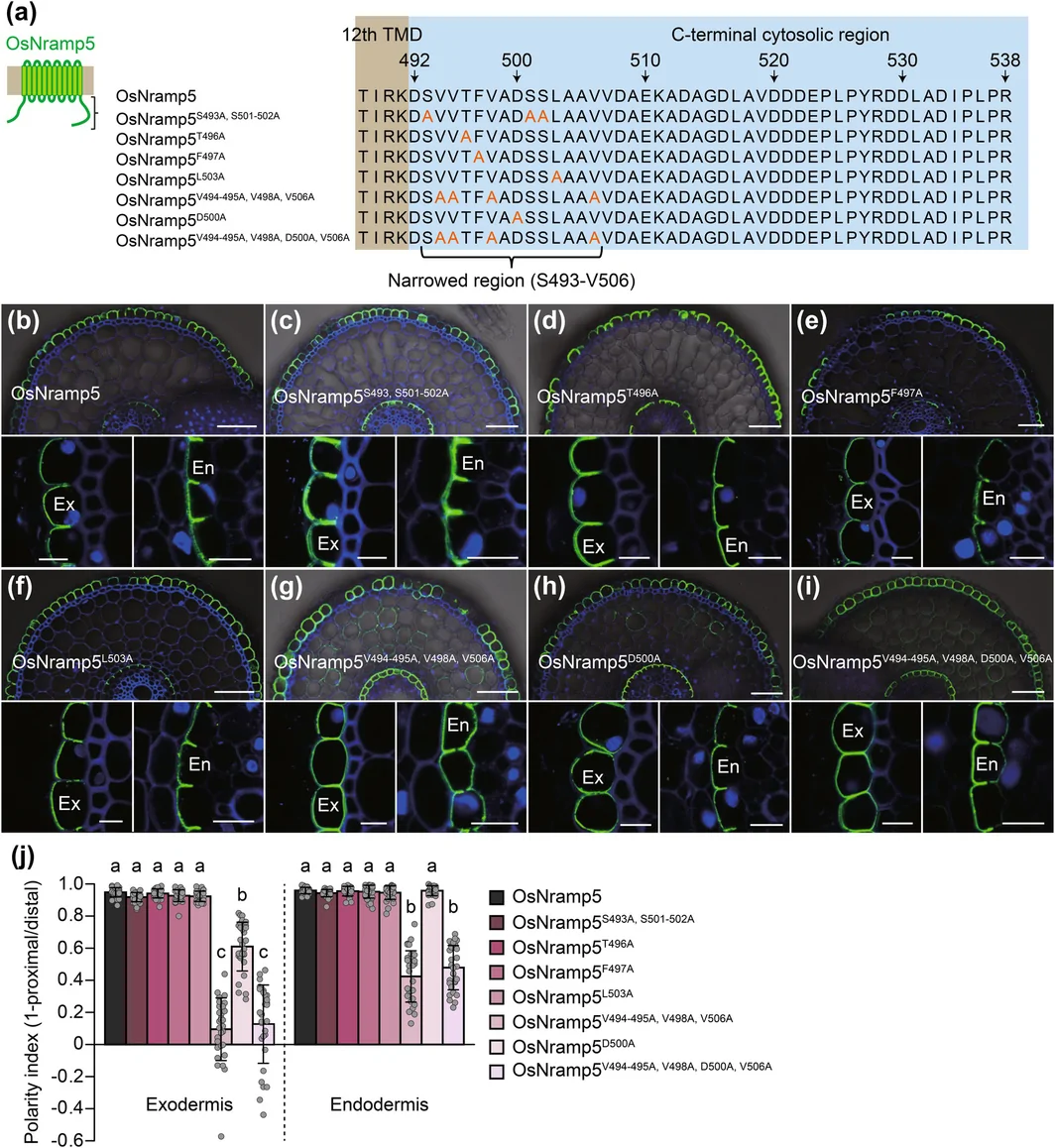
Identification of amino acid residues in the C‐terminal region required for polar localization of OsNramp5. (a) Schematic of Ala substitution sites in the C‐terminal cytosolic region of OsNramp5. Orange letters indicate residues substituted with Ala. TMD, transmembrane domain. (b–i) Localization of OsNramp5 variants: Wild‐type OsNramp5 (b), OsNramp5^S493,S501‐502A^ (c), OsNramp5^T496A^ (d), OsNramp5^F497A^ (e), OsNramp5^L503A^ (f), OsNramp5^V494‐495A,V498A,V506A^ (g), OsNramp5^D500A^ (h), OsNramp5^V494‐495A,V498A,D500A,V506A^ (i) in roots of transgenic lines. Cross‐sections of mature crown roots were immunostained with anti‐Flag antibody. Green signals represent mutated OsNramp5; blue signals indicate cell wall autofluorescence and DAPI‐stained nuclei. Enlarged images of exodermis (Ex) and endodermis (En) are shown below at each image (b–i). Scale bars: 50 μm (whole root) and 10 μm (enlarged). (j) Polarity index of OsNramp5 variants in exodermis and endodermis. Data are from 30 cells per sample from ≥ 5 root slices. Different letters indicate significant differences (*p* < 0.05, Tukey–Kramer test).

For more precise mapping, we individually substituted each amino acid from Ser493 to Val506 with Ala (Figure 4a). Substitutions at positions 493, 496, 497, 501, 502 and 503 did not affect polarity (Figure 4b–fj). In contrast, substitutions of four Val residues (Val494, Val495, Val498, Val506) strongly reduced polarity, while substitution of Asp500 caused a partial reduction (Figure 4g,h,j). These results identify four Val and one Asp as critical for polar localization. Combining mutations in all four Val with D500A did not further reduce polarity (Figure 4i,j), suggesting that these residues act in the same pathway.

### 2.6 A Val Cluster Is Required for OsNramp5 Polar Localization

To assess the contribution of individual Val, each was mutated to Ala (Figure S5a). Only the Val498A mutation partially reduced polarity; single substitutions at other Val positions had no effect (Figure S5b–f). This indicates that the Val cluster is important, with Val498 playing a more prominent role.

### 2.7 β‐Branching and High Hydrophobicity Are Required for Polarity

**FIGURE 5:**
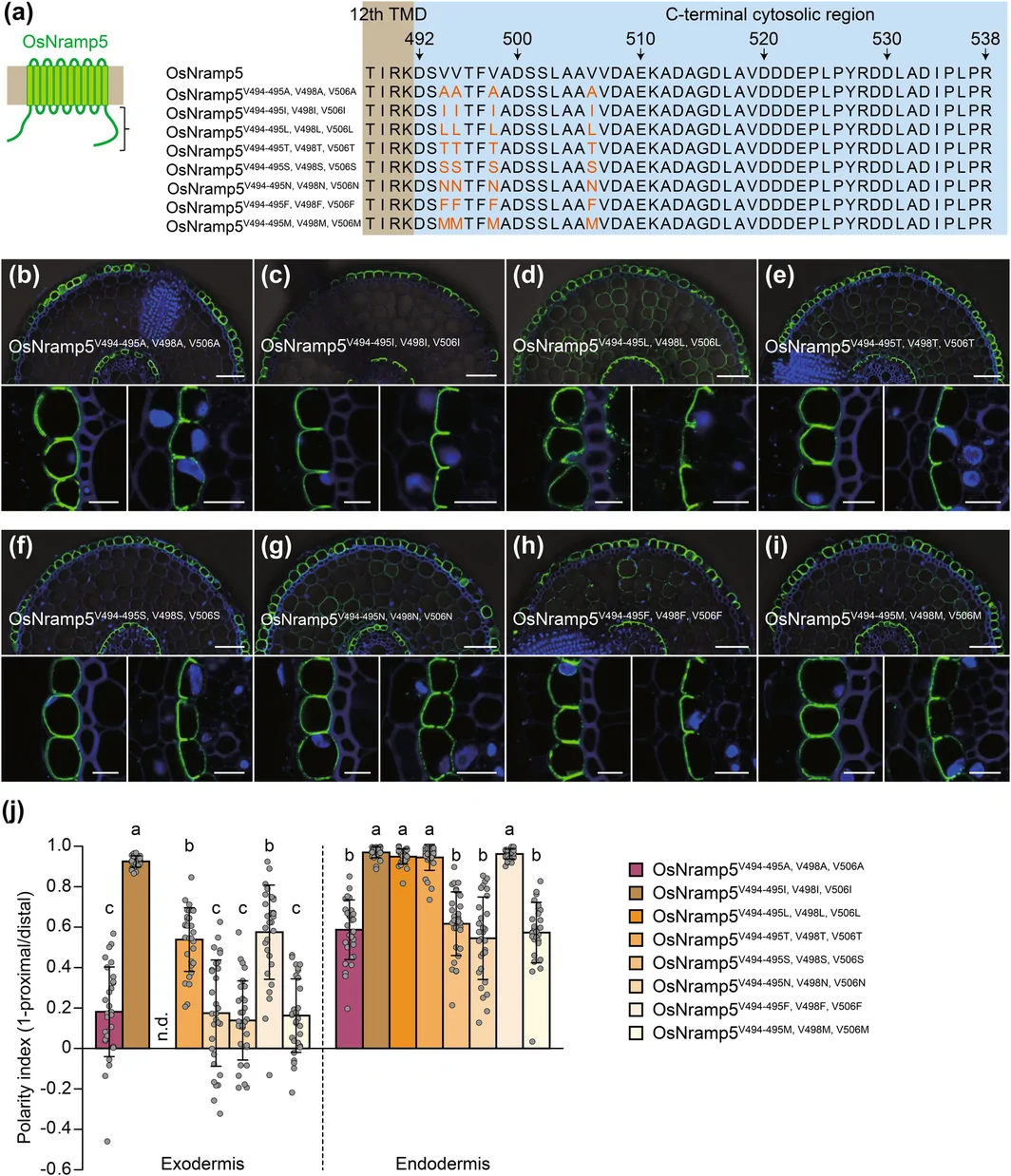
Cellular localization of OsNramp5 variants with substitutions of four Val residues. (a) Schematic of substitution sites at Val residues 494, 495, 498 and 506 in the C‐terminal region. Orange letters indicate substituted amino acids. TMD, transmembrane domain. (b–i) Localization of OsNramp5 variants with substitutions: OsNramp5^V494‐495A,V498A,V506A^ (b), OsNramp5^V494‐5I,V498I,V506I^ (c), OsNramp5^V494‐5L,V498L,V506L^ (d), OsNramp5^V494‐5T,V498T,V506T^ (e), OsNramp5^V494‐5S,V498S,V506S^ (f), OsNramp5^V494‐5N,V498N,V506N^ (g), OsNramp5^V494‐5F,V498F,V506F^ (h) and OsNramp5^V494‐5M,V498M,V506M^ (i) in roots of transgenic lines. Cross‐sections were immunostained with anti‐Flag antibody. Green signals represent mutated OsNramp5; blue signals indicate cell wall autofluorescence and DAPI‐stained nuclei. Enlarged images of exodermis (Ex) and endodermis (En) are shown below at each image (b–i). Scale bars: 50 μm (whole root) and 10 μm (enlarged). (j) Polarity index of variants in exodermis and endodermis. Data are from 30 cells per sample from ≥ 5 root slices. Different letters indicate significant differences (*p* < 0.05, Tukey–Kramer test); n.d., not determined.

To explore the biochemical properties underlying Val function, we substituted the four Val residues with various amino acids (Figure 5a). First, we substituted Val with Ile or Leu, other branched‐chain amino acids (Figure S6). Substitution with Ile fully maintained polarity in both exodermis and endodermis, unlike Ala substitution (Figure 5b,c,j). Leu substitution maintained polarity in the endodermis but caused endomembrane accumulation in the exodermis (Figure 5d,j), suggesting partial functionality.

Given that Val and Ile are β‐branched, while Leu is γ‐branched (Figure S6), we hypothesized that β‐branching is critical. To test this, we substituted Val with Thr, which has a similar structure to β‐branching (Figure S6). The Thr‐substituted variant showed partial or full polarity in the exodermis and endodermis, respectively (Figure 5e,j). In contrast, substitutions with Ser or Asn—which lack β‐branching‐like structure—resulted in non‐polar localization (Figure 5f,g,j). These results support the requirement for β‐branching at the side chain.

Since branched‐chain amino acids are highly hydrophobic, we also tested whether hydrophobicity is important. Substitution with Phe (highly hydrophobic) partially or fully maintained polarity, whereas substitution with Met (moderately hydrophobic) resulted in non‐polar localization (Figure 5h–j). Together, these findings indicate that both β‐branching and high hydrophobicity are required for OsNramp5 polarity.

### 2.8 Negative Charge of Asp500 Is Not Required for Polar Localization

**FIGURE 6:**
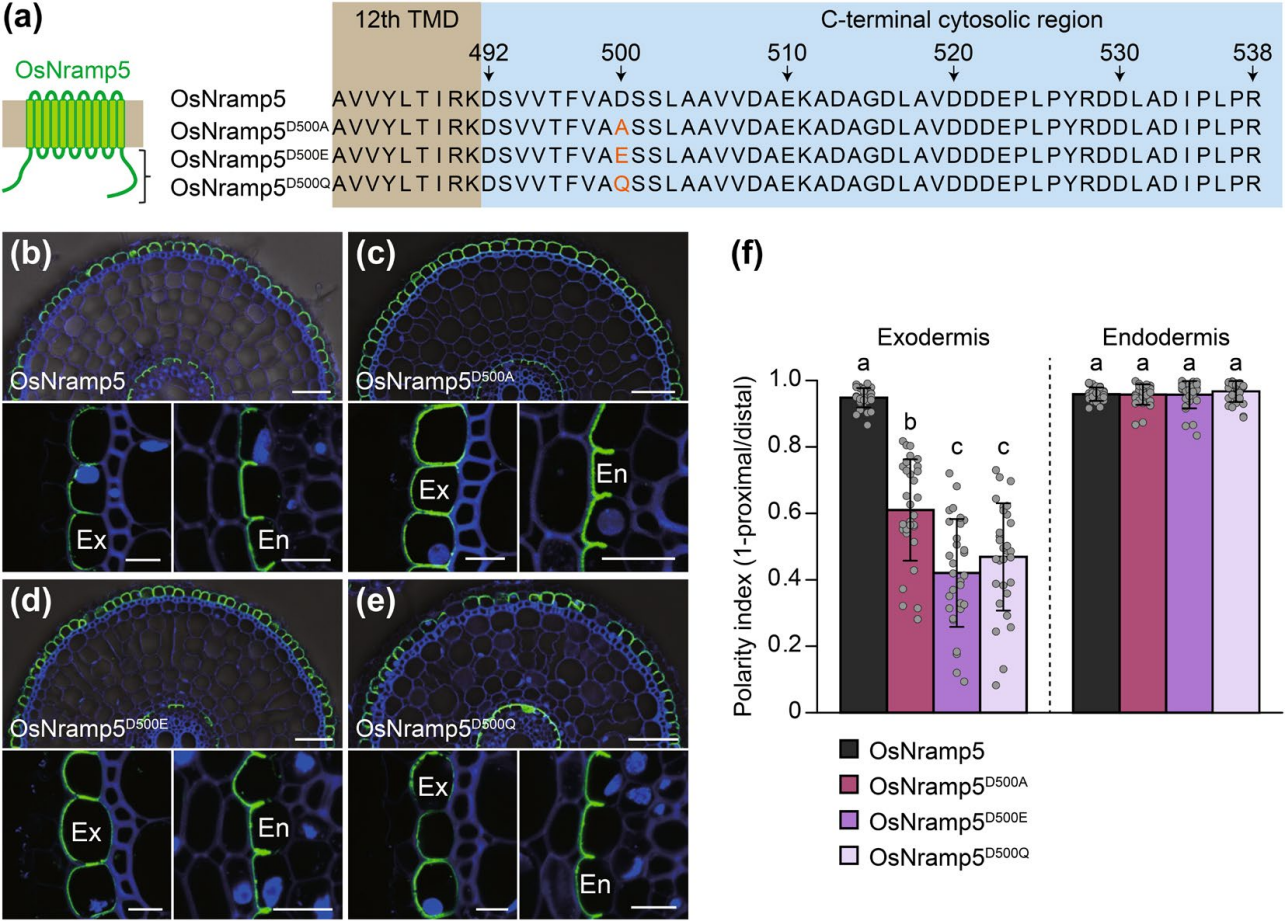
Role of the D500 residue in the polar localization of OsNramp5. (a) Schematic of D500 substitutions in the C‐terminal region. Orange letters indicate substituted amino acids. TMD, transmembrane domain. (b–e) Localization of OsNramp5 variants: Wild‐type OsNramp5 (b), OsNramp5^D500A^ (c), OsNramp5^D500E^ (d), and OsNramp5^D500Q^ (e) in roots of transgenic lines. Cross‐sections were immunostained with anti‐Flag antibody. Green signals represent mutated OsNramp5; blue signals indicate cell wall autofluorescence and DAPI‐stained nuclei. Enlarged images of exodermis (Ex) and endodermis (En) are shown below at each image (b–e). Scale bars: 50 μm (whole root) and 10 μm (enlarged). (f) Polarity index of variants in exodermis and endodermis. Data are from 30 cells per sample from ≥ 5 root slices. Different letters indicate significant differences (*p* < 0.05, Tukey–Kramer test).

To determine whether the negative charge of Asp500 is important, we substituted it with Glu (negatively charged) or Gln (neutral) (Figure 6a). Both substitutions reduced polarity to levels similar to Ala substitution (Figure 6b–f), indicating that the Asp residue itself—rather than its charge—is critical for polarity.

### 2.9 Asp500 and the Val Cluster Are Not Involved in Protein Clustering

In PIN auxin efflux carriers, protein clustering at the plasma membrane restricts lateral diffusion and maintains polarity (Li et al. 2021). To test whether similar clustering occurs for OsNramp5, we imaged the microdistribution of OsNramp5 variants at the distal side of the exodermis using super‐resolution microscopy (Figure S7a). Although OsNramp5 signal was not uniformly distributed, no obvious clustering was observed (Figure S7b,e). Moreover, the distribution patterns of D500A and Val‐cluster variants were similar to wild‐type (Figure S7c,d,f,g), suggesting that these residues are not involved in clustering.

### 2.10 Role of OsNramp5 Polar Localization in Mn Uptake

To assess the physiological relevance of polarity, we compared Mn uptake in plants expressing polarly and non‐polarly localized OsNramp5. First, we confirmed the Mn transport activity of OsNramp5 and the OsNramp5^V494‐495A,V498A,V506A^ using the Mn uptake‐deficient yeast strain, *Δsmf1*. Both variants complemented the growth defect under Mn‐limited conditions (Figure S8a) and accumulated comparable Mn levels (Figure S8b), indicating that four Val substitutions did not affect the transport activity of OsNramp5.

In transgenic rice, expression of *OsNramp5* variants under the *OsLsi1* promoter was ~20‐fold higher than endogenous levels, but comparable across transgenic lines and Mn conditions (Figure S9a). Expression of *OsMTP9* was also similar across lines (Figure S9b). Under low (0.5 μM) Mn conditions, all variants complemented the growth defect of the *OsNramp5* knockout, though complementation was not always complete (Figure S10a,b), likely due to the absence of *OsNramp5* expression in leaf sheaths (Huang et al. 2024). Under high Mn (5 μM), growth was similar to the mutant in all lines (Figure S10a,b).

**FIGURE 7:**
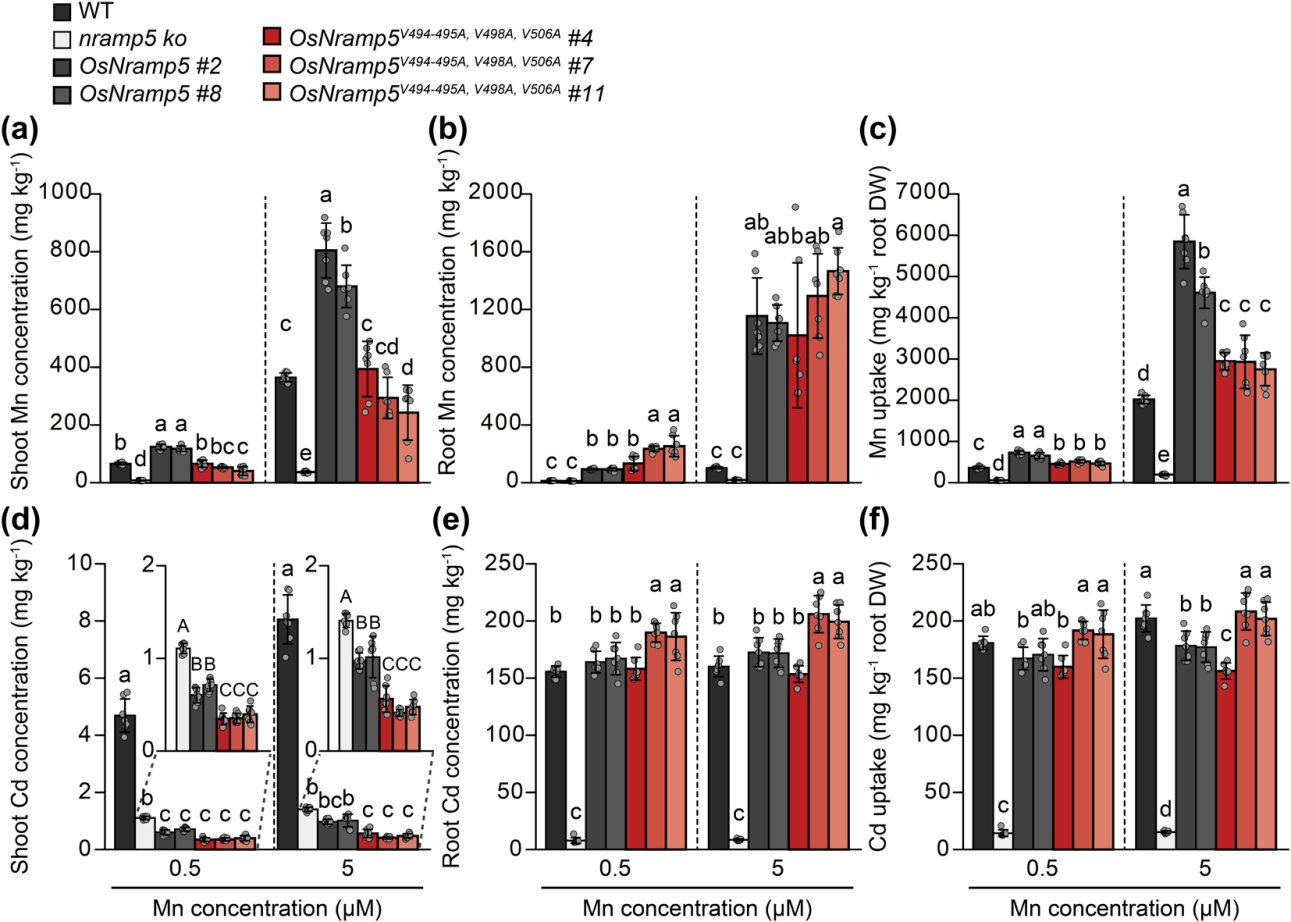
Role of OsNramp5 polarity in Mn and Cd uptake. (a–f) Concentration of Mn (a, b) or Cd (d, e) in shoots (a, d) and roots (b, e), and total uptake of Mn (c) or Cd (f) in wild‐type (WT), *nramp5* loss‐of‐function mutant, and transgenic lines expressing polarly (*OsNramp5‐Flag*) or non‐polarly (*OsNramp5* ^*V494‐495A*,*V498A*,*V506A*^ *‐Flag*) localized *OsNramp5*. Seedlings were grown in a nutrient solution containing 0.5 or 5 μM Mn for 8 days, followed by exposing to 0.5 μM Cd for 1 day with 0.5 or 5 μM Mn. Shoots and roots were harvested separately. Mn and Cd concentrations were determined by ICP‐MS. Uptake was calculated as (shoot metal content + root metal content)/root dry weight. Data are means ± SD (*n* = 7). Different small letters indicate significant differences across all lines, while different large letters indicate significant differences across all lines except WT (*p* < 0.05, Tukey–Kramer test).

Mn accumulation analysis revealed that under both low and high Mn conditions, plants expressing non‐polarly localized OsNramp5 had significantly lower shoot Mn concentrations than those expressing polarly localized OsNramp5 (Figure 7a). Root Mn concentrations were either increased or unchanged in non‐polar lines (Figure 7b), and total Mn uptake followed the same trend as shoot Mn (Figure 7c). These results demonstrate that polar localization of OsNramp5 is essential for efficient Mn uptake.

### 2.11 Role of OsNramp5 Polar Localization in Cd Uptake

OsNramp5 is able to transport both Mn and Cd (Sasaki et al. 2012). We therefore examined whether OsNramp5 polarity also affects Cd uptake. After exposure to 0.5 μM Cd for 1 day in the presence of 0.5 or 5 μM Mn, similar to Mn, the plants carrying non‐polarly localized OsNramp5 showed lower shoot Cd concentration than the plants carrying polarly localized OsNramp5 at either Mn concentration (Figure 7d). In the roots, there was no large difference in Cd concentration between plants carrying non‐polarly and polarly localized OsNramp5 (Figure 7e). Interestingly, the shoot Cd concentration in the transgenic plants carrying either polarly or non‐polarly localized *OsNramp5* was even lower than that of the *nramp5* mutant, although the total Cd uptake of these transgenic lines was compensated to the WT levels (Figure 7d–f).

## 3 Discussion

In the present study, we identified amino acid residues critical for polar localization of OsNramp5 through detailed site‐directed mutagenesis and immunostaining. We also provide experimental evidence that polar localization is required for efficient Mn uptake by comparing transgenic plants expressing polarly and non‐polarly localized OsNramp5.

### 3.1 The C‐Terminal Cytosolic Region, but Not AP2‐Dependent CME, Is Required for OsNramp5 Polarity

In Arabidopsis, the AP2‐dependent CME is required for the lateral polarity of mineral transporters, such as AtNIP5;1, AtBOR1 and AtIRT1 (Wang et al. 2017; Yoshinari et al. 2016, 2019; Spielmann et al. 2025). In contrast, in rice, the AP2‐dependent CME is not required for the polar localization of mineral transporters tested, such as OsLsi1, OsLsi2 and OsBOR1 (Huang, Konishi, et al. 2022; Konishi et al. 2022). In the present study, we found that AP2‐depedent CME is also not required for polar localization of OsNramp5 (Figure 1). These findings indicate that the mechanisms underlying the polar localization of mineral transporters differ between rice and Arabidopsis.

Instead of endocytosis, polar localization of transporters may include polar exocytosis and/or anchoring mechanisms to limit diffusion of transport proteins in the plasma membrane (Kleine‐Vehn et al. 2011; Łangowski et al. 2016). In the present study, we revealed that the C‐terminal, but not the N‐terminal, region of OsNramp5 is necessary and sufficient to give polarity for OsNramp members (Figures 2 and 3, Figure S1). More detailed analysis showed that the four Val (494, 495, 498, 506) and Asp500 residues are crucial for polar localization (Figure 4). Furthermore, the β‐branching and high hydrophobicity of Val residues, but not the negative charge of Asp500, are required for its polarity (Figures 5 and 6). When comparing the amino acid sequences of OsNramp members, four Val and Asp500 residues are not conserved in the non‐polarly localized homologues, including OsNramp1, OsNramp3 and OsNramp4 (Figure S11), further supporting the importance of these residues for the polar localization. Since the C‐terminus of OsNramp5 is exposed to the cytoplasm, these residues are likely required for interaction with unknown cytosolic polarity factor(s). Interestingly, Ile, another β‐branching and hydrophobic amino acid, located in the cytosolic tails of OsLsi1, is also required for its polarity (Konishi et al. 2023), suggesting a common mechanism for generating distal polarity in rice roots. Although we have no idea why the β‐branching of Val residues is required, their hydrophobicity may be involved in the hydrophobic bonding with the polarity factor. In Arabidopsis, apical‐basal polarity of PIN proteins are maintained by protein clustering to limit their diffusion, together with endocytosis (Marhava 2022). However, the substitutions of Val and Asp residues have less effect on the micro‐localization of OsNramp5 at the distal side of the exodermis cell (Figure S7), indicating that these residues are unlikely to be involved in anchoring processes by making protein clustering. Therefore, these residues may be involved in polar exocytosis. During exocytosis, the exocyst complex has a role in tethering secretory vesicles to specific sites on the plasma membrane, facilitating the subsequent vesicle‐membrane fusion mediated by the SNARE proteins (Žárský 2022). In fact, one of the exocyst components, AtEXO84b, showed polar localization on the distal side in epidermal cells of Arabidopsis roots, and its knockout impaired the distal polarity of PENETRATION3, an ATP‐binding cassette transporter involved in defence against some fungal pathogens (Mao et al. 2016). Four Val residues and Asp500 identified may be related to these processes although the exact mechanisms need to be investigated in the future.

### 3.2 Polarity of OsNramp5 Is Required for Efficient Mn Uptake

Mn uptake in rice roots is mediated by two different transporters; OsNramp5 and OsMTP9 (Sasaki et al. 2012; Ueno et al. 2015). OsNramp5 functions as an influx transporter of Mn, while OsMTP9 functions as an efflux transporter. Both of OsNramp5 and OsMTP9 are polarly localized at the distal and proximal side, respectively, of exodermis and endodermis (Sasaki et al. 2012; Ueno et al. 2015), where Casparian strips are located (Enstone et al. 2002). Therefore, OsNramp5 and OsMTP9 are proposed to form an efficient and directional system for Mn uptake (Figure S12a; Shao et al. 2017). In the present study, we showed experimental evidence on important role of polar localization of OsNramp5 in Mn uptake; loss of OsNramp5 polarity significantly reduced Mn uptake under both low and high Mn conditions (Figure 7). This effect could be attributed to re‐uptake of Mn from the proximal side of the apoplast (aerenchyma) (Figure S12b), decreasing directional transport of Mn from soil to the stele for subsequent translocation of Mn to the shoots. These findings are consistent with studies on other polarized transporters, such as AtNIP5;1 (Wang et al. 2017), AtIRT1 (Spielmann et al. 2025), and OsLsi1 (Konishi et al. 2023, 2025), underscoring the general importance of polarity for efficient mineral uptake.

Some transporters for mineral elements do not show polar localization. For example, HvNramp5 involved in Mn uptake in barley is localized in the root epidermal cells without polarity (Wu et al. 2016). In the future, there is a potential to manipulate these transporters to be polarly localized to enhance efficiency of mineral element uptake, especially under nutrient‐limited conditions.

### 3.3 Potential Application of OsNramp5 Polarity in Reducing Cd Accumulation

Rice is a staple food and a major dietary source of Cd, a toxic heavy metal (Shimbo et al. 2001). Therefore, reducing Cd accumulation in rice grains is an important issue for human health. OsNramp5 is a key transporter for both Mn and Cd (Sasaki et al. 2012; Ishikawa et al. 2012). However, simple knockout of *OsNramp5* decreases not only Cd accumulation but also Mn accumulation, leading to growth defects (Sasaki et al. 2012). Indeed, in the present study, we also observed inhibited growth of an *OsNramp5* knockout line under both low and high Mn concentrations (Figure S10).

Unexpectedly, we found that Cd accumulation in the shoots of transgenic lines expressing polarly and non‐polarly localized OsNramp5 was even lower than that of the *nramp5* knockout line (Figure 7d), while their growth was similar to that of the wild‐type rice (Figure S10). This result may be attributed to *OsNramp5* overexpression. In this study, we used the *OsLsi1* promoter to drive *OsNramp5* expression in the root exodermis and endodermis. The *OsLsi1* promoter exhibits high transcriptional activity, as evidenced by the elevated expression level of *OsNramp5* in roots (Figure S9a). Increased *OsNramp5* expression facilitates Mn influx into root cells, thereby reducing root‐to‐shoot translocation, likely through competition between Mn and Cd for xylem loading, although the Cd efflux transporter had not been identified. This finding is consistent with previous studies using the *OsActin1* promoter (Chang, Huang, Konishi, et al. 2020; Chang, Huang, Yamaji, et al. 2020) or natural duplication of *OsNramp5* (Yu et al. 2022).

Furthermore, we observed that plants carrying non‐polarly localized OsNramp5 accumulated less Cd in shoots compared to those carrying polarly localized OsNramp5 (Figure 7d). Similar to the pattern observed for Mn (Figure S12), this may be explained by reduced Cd uptake efficiency due to loss of OsNramp5 polarity. Notably, given that Mn concentration was complemented to WT levels and growth was restored in plants expressing non‐polarly localized OsNramp5 due to its overexpression, this approach may offer an alternative strategy for reducing Cd accumulation in rice grains; although further studies are required for agronomical application.

In conclusion, we identified a cluster of four valine residues (Val494, Val495, Val498 and Val506) along with Asp500 as critical for the polar localization of OsNramp5. The requirement for β‐branching and high hydrophobicity at these valine positions highlights the structural basis of this polarity. Moreover, we demonstrate that polar localization is physiologically essential for efficient Mn uptake in rice and holds potential for future applications in reducing Cd accumulation.

## 4 Materials and Methods

### 4.1 Generation of Transgenic Lines Harbouring *OsNramp5* Variants and Other *OsNramp* Genes

Coding sequences (CDSs) of *OsNramp5* (Os07g0257200), *OsNramp1* (Os07g0258400), *OsNramp3* (Os06g0676000) and *OsNramp4* (Os02g0131800) were amplified from root cDNA of rice (cv. Nipponbare) by PCR, and a Flag tag was fused to the C‐terminus via overlapping PCR. Chimeric genes were constructed by swapping regions between OsNramp5 and its homologues using overlapping PCR. Site‐directed mutagenesis was performed similarly to generate C‐terminal substitution variants. Plasmids containing these CDSs were cloned downstream of the *OsLsi1* promoter (2000 bp) in the pPZP2H‐lac vector using HindIII and XbaI sites via In‐Fusion cloning (Takara). A *nopaline synthase* terminator was inserted using the SacI site. Constructs were transformed into calluses of the *OsNramp5* loss‐of‐function mutant (lcd‐kmt2) (Ishikawa et al. 2012) via *Agrobacterium*‐mediated transformation (Hiei and Komari 2008). Primers and templates are listed in Table S1.

### 4.2 Immunostaining Analysis

Knockout mutants of the *AP2M* gene were generated in our previous study (Huang, Konishi, et al. 2022). Homo mutated plants (−/−) and nonmutated plants (+/+) were selected from the heterozygous seedlings by genomic PCR (Huang, Konishi, et al. 2022).

Root samples were collected from transgenic seedlings carrying various OsNramp variants at the 6–7 leaf stage (T0 or T1). T1 seeds were germinated in water at 30°C for 2 days, transferred to 0.5 mM CaCl_2_, and then to half‐strength Kimura B solution (Ma et al. 2002), changed every 2 days. Plants were grown in a greenhouse at 25°C–30°C under natural light. Immunostaining was performed as described (Ma et al. 2006; Konishi and Ma 2021). Cross‐sections of mature crown roots (15–20 mm from tip) were prepared with a MicroSlicer (LinearSlicer PRO10, Dosaka EM). For Flag or OsNramp5 detection, a rat monoclonal anti‐Flag antibody (L5, 1/1000, Novus Biologicals) or a rabbit polyclonal anti‐OsNramp5 antibody (1/500, Sasaki et al. 2012) was used, respectively. Double staining for Flag and an ER marker used mixed primary antibodies (anti‐Flag and monoclonal anti‐HDEL, 2E7, 1/500, Santa Cruz). Alexa Fluor 555 goat anti‐rat IgG, Alexa Fluor 555 goat anti‐rabbit IgG or Alexa Fluor 488 goat anti‐mouse IgG (Thermo Fisher) was used as a secondary antibody to label Flag, OsNramp5, or HDEL antibody, respectively. Nuclei were stained with DAPI. Fluorescence was imaged with a confocal microscope (TCS SP8x, Leica). Signal intensity was quantified using LAS AF Lite software (Leica) as described (Konishi et al. 2023). The polarity index was calculated as (1—distal/proximal signal ratio) for each exodermal and endodermal cell.

### 4.3 Super‐Resolution Microscopy

To examine OsNramp5 distribution, 23‐day‐old plants expressing Flag‐tagged variants were used. Tilted root sections (~30°) were prepared and immunostained as above. Super‐resolution imaging was performed using LIGHTNING mode (Leica) on a 63× oil immersion lens. *Z*‐stacks (4.5 μm depth, 17 slices at 0.28 μm intervals) were acquired and merged.

### 4.4 Mn Transport Assay in Yeast

CDSs of *OsNramp5‐Flag* and *OsNramp5* ^*V494‐495A*,*V498A*,*V506A*^ *‐Fla*g were cloned into pDR196 (EcoRI/SalI) via In‐Fusion. Plasmids or empty vector were introduced into the *Δsmf1* yeast mutant (Portnoy et al. 2000). For growth assays, yeast was spotted on SD‐Uracil plates with or without 5 mM EGTA. For Mn uptake quantification, yeast was cultured in SD‐Uracil with 4.6 μM Mn for 5.5 h, collected, washed, dried, digested in HNO₃, and analysed by ICP‐MS (7700X, Agilent).

### 4.5 RNA Extraction and qRT‐PCR

Roots from Mn uptake experiments were harvested (four replicates). RNA was extracted using the RNeasy Plant Mini Kit (Qiagen), and cDNA was synthesized with ReverTra Ace qPCR RT Master Mix (Toyobo). Expression of *OsNramp5* and *OsMTP9* was measured using KOD SYBR qPCR Mix (Toyobo) on a CFX384 system (Bio‐Rad) with primers from Sasaki et al. (2012) and Ueno et al. (2015). *HistoneH3* and *Ubiquitin* served as internal controls (Konishi et al. 2025). Relative expression was calculated via the comparative Ct method.

### 4.6 Mn and Cd Uptake Comparison in Plants

Wild‐type (WT, cv. Koshihikari), *OsNramp5* loss‐of‐function mutant, and transgenic lines expressing polarly or non‐polarly localized OsNramp5 were germinated as above. At day 6, seedlings were transferred to half‐strength Kimura B with 0.5 μM Mn. At day 11, plants were exposed to 0.5 or 5 μM Mn for 8 days, followed by exposing to 0.5 μM Cd with 0.5 or 5 μM Mn for 1 day (seven replicates per treatment). Roots were washed with 5 mM CaCl_2_, separated from shoots, dried, digested in HNO_3_ and analysed by ICP‐MS.

### 4.7 Statistical Analysis

ANOVA with Tukey–Kramer post hoc test was performed using BellCurve for Excel (Social Survey Research Information Co. Ltd).

## Author Contributions

N.K. and J.F.M. conceived and designed the experiments; N.K. performed the experiments. N.K. and J.F.M. analysed data and wrote the manuscript.

## Funding

This work was supported by Japan Society for the Promotion of Science, 21H05034, 25H01332, 26K21758, 24K17807, 26K08575, 26H01730 and JPJS00420230010.

## Conflicts of Interest

The authors declare no conflicts of interest.

## Supporting information


**Figure S1:** Cellular localization of OsNramp5 in *ap2m* mutants.
**Figure S2:** Cellular localization of chimeric proteins generated by swapping N‐terminal regions between OsNramp5 and its
**Figure S3:** Cellular localization of OsNramp5 variants with Ala substitutions in the D492–V506 region.
**Figure S4:** ER localization of OsNramp5^D492A^.
**Figure S5:** Cellular localization of OsNramp5 variants with single Val substitutions.
**Figure S6:** Structure and properties of amino acid side chains used for substitutions.
**Figure S7:** Protein clustering analysis of polarly, weak‐polarly, and non‐polarly localized OsNramp5 variants in the exodermis.
**Figure S8:** Mn transport activity of OsNramp5 variants in yeast.
**Figure S9:** Expression of Mn transporters in roots of transgenic plants expressing polarly or non‐polarly localized *OsNramp5*.
**Figure S10:** Growth of plants expressing polarly or non‐polarly localized *OsNramp5* under 0.5 and 5 μM Mn conditions.
**Figure S11:** Amino acid sequence alignment of the C‐terminal region of polar and non‐polar OsNramp members.
**Figure S12:** Schematic model of the role of OsNramp5 polar localization in Mn uptake in rice.

**Table S1:** Primer sequences and templates for the construction of various variants of *OsOsNramp5*, *OsNramp1*, *OsNramp3* and *OsNramp4*

## Data Availability

The experiment data that support the findings of this study is available from the corresponding author upon reasonable request.
