# Supplementary material for: Identification of Critical Amino Acid Residues Required for the Polar Localization of a Rice Manganese Transporter
Source: Plant Biotechnol J. 2026 Jun 25:10.1111/pbi.70708. Online ahead of print. doi: 10.1111/pbi.70708 (PMC13399035; doi:10.1111/pbi.70708)
Supplement: Supplementary file 1 — Figure S1: Cellular localization of OsNramp5 in ap2m mutants. Figure S2: Cellular localization of chimeric proteins generated by swapping N‐terminal regions between OsNramp5 and its Figure S3: Cellular localization of OsNramp5 variants with Ala substitutions in the D492–V506 region. Figure S4: ER localization of OsNramp5D492A. Figure S5: Cellular localization of OsNramp5 variants with single Val substitutions. Figure S6: Structure and properties of amino acid side chains used for substitutions. Figure S7: Protein clustering analysis of polarly, weak‐polarly, and non‐polarly localized OsNramp5 variants in the exodermis. Figure S8: Mn transport activity of OsNramp5 variants in yeast. Figure S9: Expression of Mn transporters in roots of transgenic plants expressing polarly or non‐polarly localized OsNramp5. Figure S10: Growth of plants expressing polarly or non‐polarly localized OsNramp5 under 0.5 and 5 μM Mn conditions. Figure S11: Amino acid sequence alignment of the C‐terminal region of polar and non‐polar OsNramp members. Figure S12: Schematic model of the role of OsNramp5 polar localization in Mn uptake in rice. [file PBI-9999-0-s002.pdf]

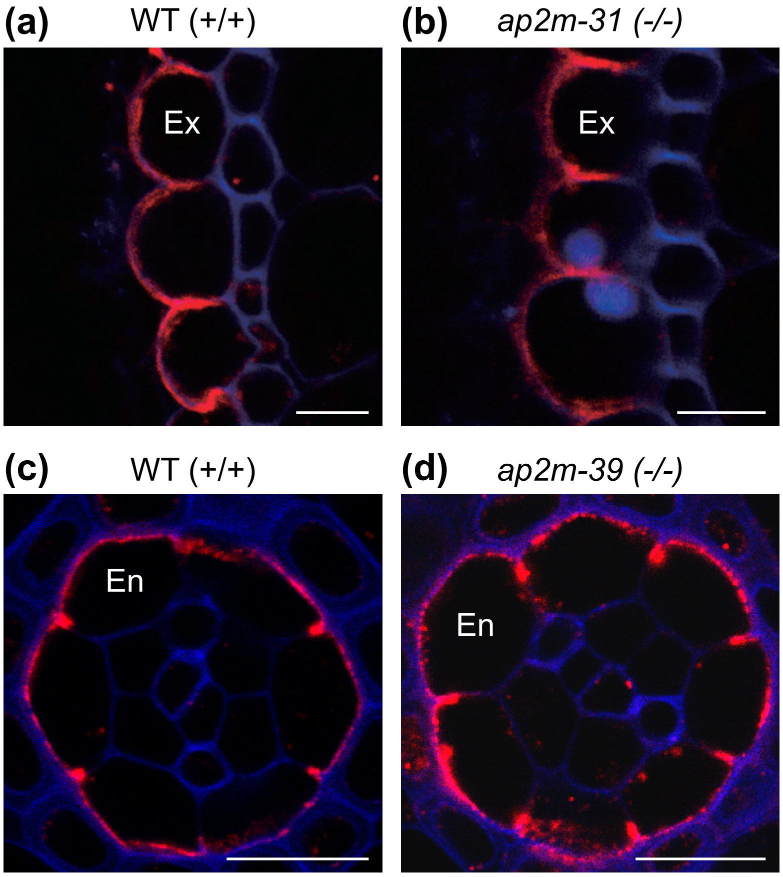

Figure S1. Cellular localization of OsNramp5 in *ap2m* mutants.

(a-d) Localization of OsNramp5 in the exodermis (Ex) of crown roots (a-b) and in the endodermis (En) of lateral roots (c-d) in WT (a, c) and knockout lines of the *OsAP2M* gene (b, c). WT (+/+) and knockout lines (−/−) *(ap2m-31, −39*) were selected from a heterozygous population of CRISPR/Cas9-mediated mutant lines. Crown roots (10-20 mm from the root tip) and lateral roots of the seedlings (3-week-old) were used for immunostaining using antibodies of OsNramp5. Red signals represent OsNramp5; blue signals indicate cell wall autofluorescence and DAPI-stained nuclei. Bars indicate 10 µm.

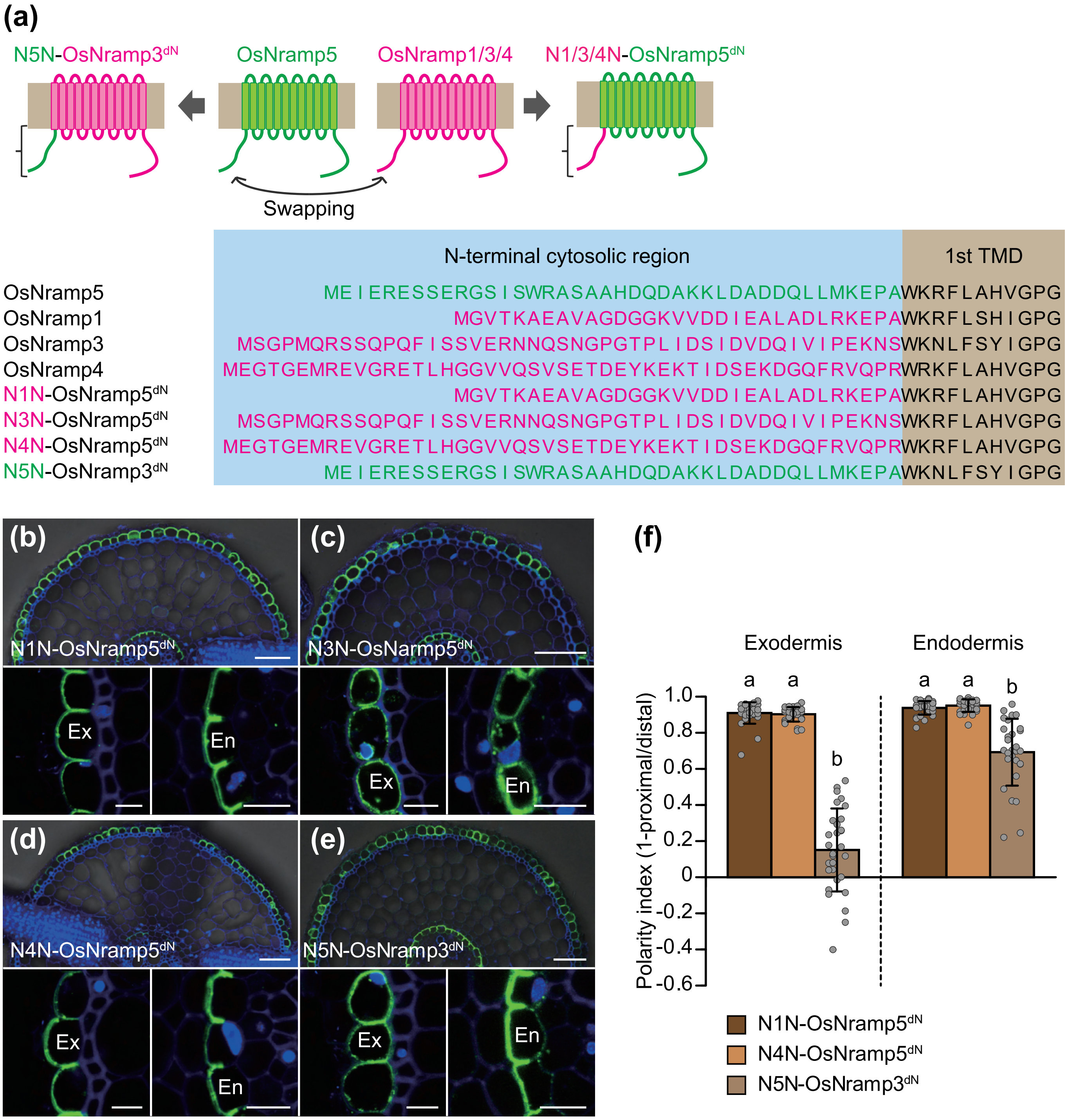

Figure S2. Cellular localization of chimeric proteins generated by swapping N-terminal regions between OsNramp5 and its homologs.

(a) Schematic representation of chimeric protein design. Green indicates OsNramp5 topology; magenta indicates topology of OsNramp1 OsNramp3, or OsNramp4. Magenta sequences represent swapped regions from homologs; green sequences are from OsNramp5. TMD, transmembrane domain. (b-e) Localization of chimeric proteins: N1N-OsNramp5^{dN} (b), N3N-OsNramp5^{dN} (c), N4N-OsNramp5^{dN} (d), or N5N-OsNramp3^{dN} (e) in roots of transgenic plants. Cross-sections were immunostained. Green signals represent chimeric proteins; blue signals indicate cell wall autofluorescence and DAPI-stained nuclei. Enlarged images of exodermis (Ex) and endodermis (En) are shown below. Scale bars: 50 μm (whole root) and 10 μm (enlarged). (f) Polarity index of chimeric proteins in exodermis and endodermis. Data are from 30 cells per sample from ≥5 root slices. Different letters indicate significant differences ($P < 0.05$, Tukey–Kramer test).

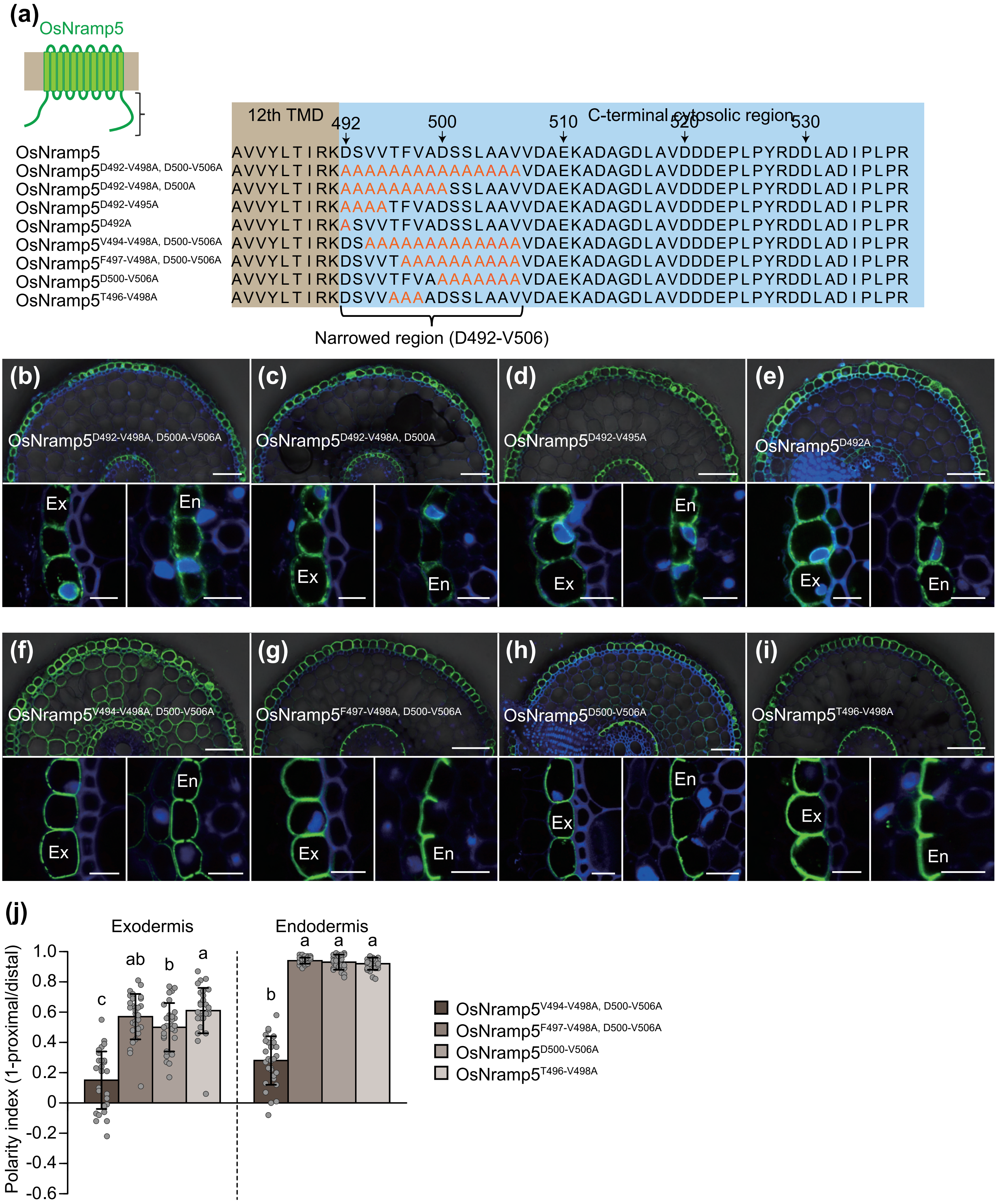
(a)
OsNramp5
12th TMD
C-terminal cytosolic region
492
500
510
520
530
OsNramp5 AVVYLTIRKDSVVTFVADSSLAAVVDAEKADAGDLAVDDDEPLPYRDDLADIPLPR
OsNramp5D492-V498A, D500-V506A AVVYLTIRKAAAAAAAAAAAAAAAVDAEKADAGDLAVDDDEPLPYRDDLADIPLPR
OsNramp5D492-V498A, D500A AVVYLTIRKAAAAAAAAASSLAAVVDAEKADAGDLAVDDDEPLPYRDDLADIPLPR
OsNramp5D492-V495A AVVYLTIRKAAAATFVADSSLAAVVDAEKADAGDLAVDDDEPLPYRDDLADIPLPR
OsNramp5D492A AVVYLTIRKASVVTFVADSSLAAVVDAEKADAGDLAVDDDEPLPYRDDLADIPLPR
OsNramp5V494-V498A, D500-V506A AVVYLTIRKDSAAAAAAAAAAAAAVDAEKADAGDLAVDDDEPLPYRDDLADIPLPR
OsNramp5F497-V498A, D500-V506A AVVYLTIRKDSVVTAAAAAAAAAAVDAEKADAGDLAVDDDEPLPYRDDLADIPLPR
OsNramp5D500-V506A AVVYLTIRKDSVVTFVAAAAAAAAVDAEKADAGDLAVDDDEPLPYRDDLADIPLPR
OsNramp5T496-V498A AVVYLTIRKDSVVAAAADSSLAAVVDAEKADAGDLAVDDDEPLPYRDDLADIPLPR
Narrowed region (D492-V506)
(b) OsNramp5D492-V498A, D500A-V506A
(c) OsNramp5D492-V498A, D500A
(d) OsNramp5D492-V495A
(e) OsNramp5D492A
(f) OsNramp5V494-V498A, D500-V506A
(g) OsNramp5F497-V498A, D500-V506A
(h) OsNramp5D500-V506A
(i) OsNramp5T496-V498A
Ex
En
(j)
Exodermis
Endodermis
Polarity index (1-proximal/distal)
1.0
0.8
0.6
0.4
0.2
0
-0.2
-0.4
-0.6
c
ab
b
a
b
a
a
a
OsNramp5V494-V498A, D500-V506A
OsNramp5F497-V498A, D500-V506A
OsNramp5D500-V506A
OsNramp5T496-V498A

Figure S3. Cellular localization of OsNramp5 variants with Ala substitutions in the D492–V506 region.

(a) Schematic of substitution sites in the C-terminal cytosolic region. Orange letters indicate residues substituted with Ala. TMD, transmembrane domain. (b-i) Localization of OsNramp5 variants: OsNramp5$^{D492A\text{-}V498A,\ D500A\text{-}V506A}$ (b), OsNramp5$^{D492A\text{-}V498A,\ D500A}$ (c), OsNramp5$^{D492A\text{-}V495A}$ (d), OsNramp5^{D492A} (e), OsNramp5$^{V494A\text{-}V498A,\ D500A\text{-}V506A}$ (f), OsNramp5$^{F497A\text{-}V498A,\ D500A\text{-}V506A}$ (g), OsNramp5$^{D500A\text{-}V506A}$ (h), and OsNramp5$^{T496A\text{-}V498A}$ (i) in roots of transgenic lines. Cross-sections were immunostained with anti-Flag antibody. Green signals represent mutated OsNramp5; blue signals indicate cell wall autofluorescence and DAPI-stained nuclei. Enlarged images of exodermis (Ex) and endodermis (En) are shown below. Scale bars: 50 µm (whole root) and 10 µm (enlarged). (j) Polarity index of variants in exodermis and endodermis. Data are from 30 cells per sample from ≥5 root slices. Different letters indicate significant differences ($P < 0.05$, Tukey–Kramer test).

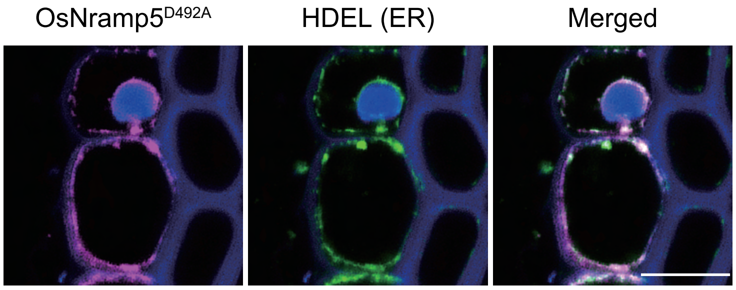

Figure S4. ER localization of OsNramp5^{D492A}.

Double immunostaining of OsNramp5^{D492A} and the ER marker HDEL in cross-sections of mature crown roots from transgenic lines expressing *OsNramp5^{D492A}-Flag*. Magenta signals represent anti-Flag staining; green signals represent anti-HDEL staining; blue signals indicate cell wall autofluorescence and DAPI-stained nuclei. Enlarged view of exodermis cells is shown. Scale bar: 10 µm.

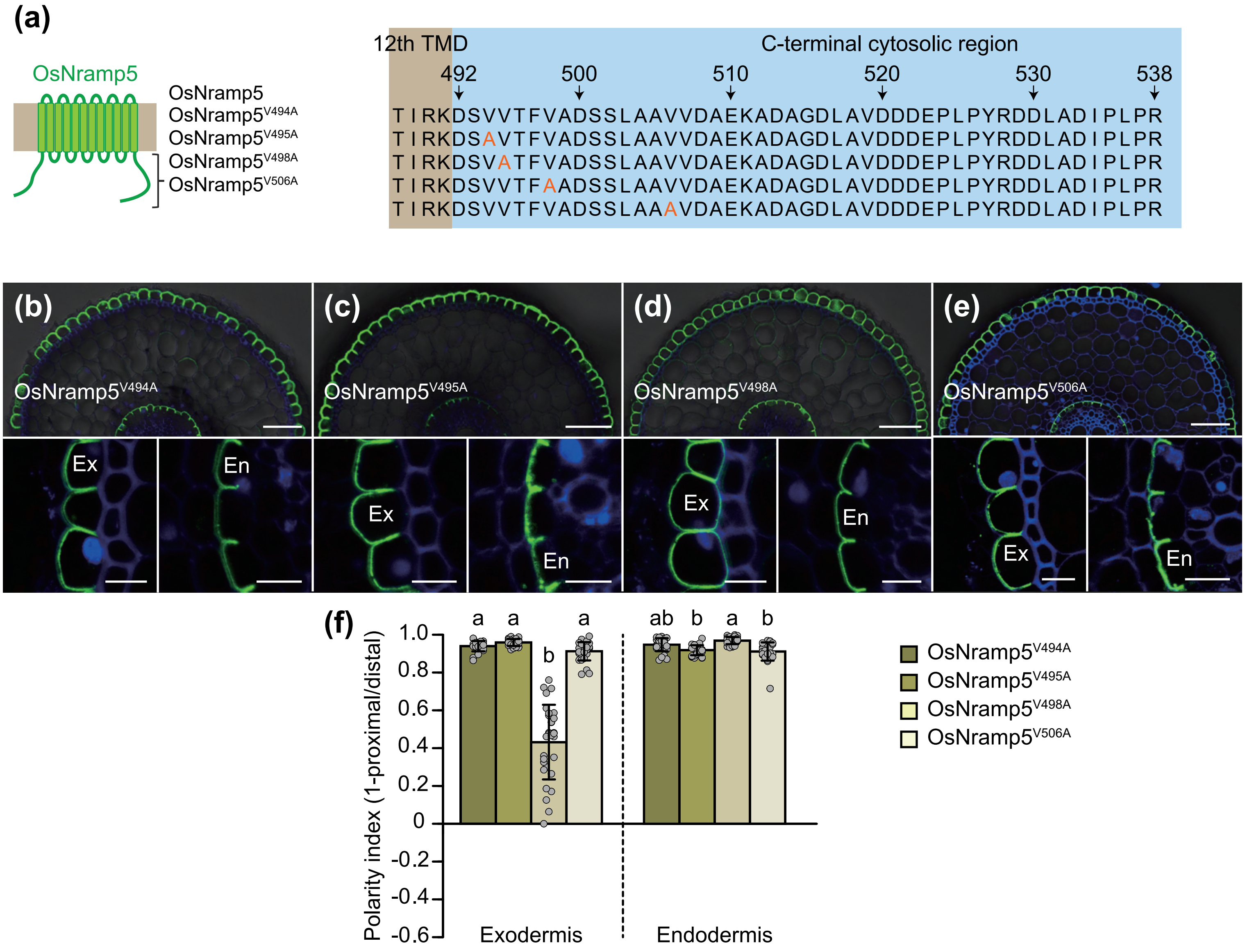

Figure S5. Cellular localization of OsNramp5 variants with single Val substitutions.

(a) Schematic of single Val substitution sites. Orange letters indicate residues substituted with Ala. TMD, transmembrane domain. (b-e) Localization of OsNramp5 variants: OsNramp5^{V494A} (b), OsNramp5^{V495A} (c), OsNramp5^{V498A} (d), and OsNramp5^{V506A} (e) in roots of transgenic lines carrying mutated *OsNramp5-Flag*. Cross-sections were immunostained with anti-Flag antibody. Enlarged images of exodermis (Ex) and endodermis (En) are shown below. Green signals represent mutated OsNramp5; blue signals indicate cell wall autofluorescence and DAPI-stained nuclei. Scale bars: 50 µm (whole root) and 10 µm (enlarged). (f) Polarity index of variants in exodermis and endodermis. Data are from 30 cells per sample from ≥5 root slices. Different letters indicate significant differences ($P <$ 0.05, Tukey–Kramer test).

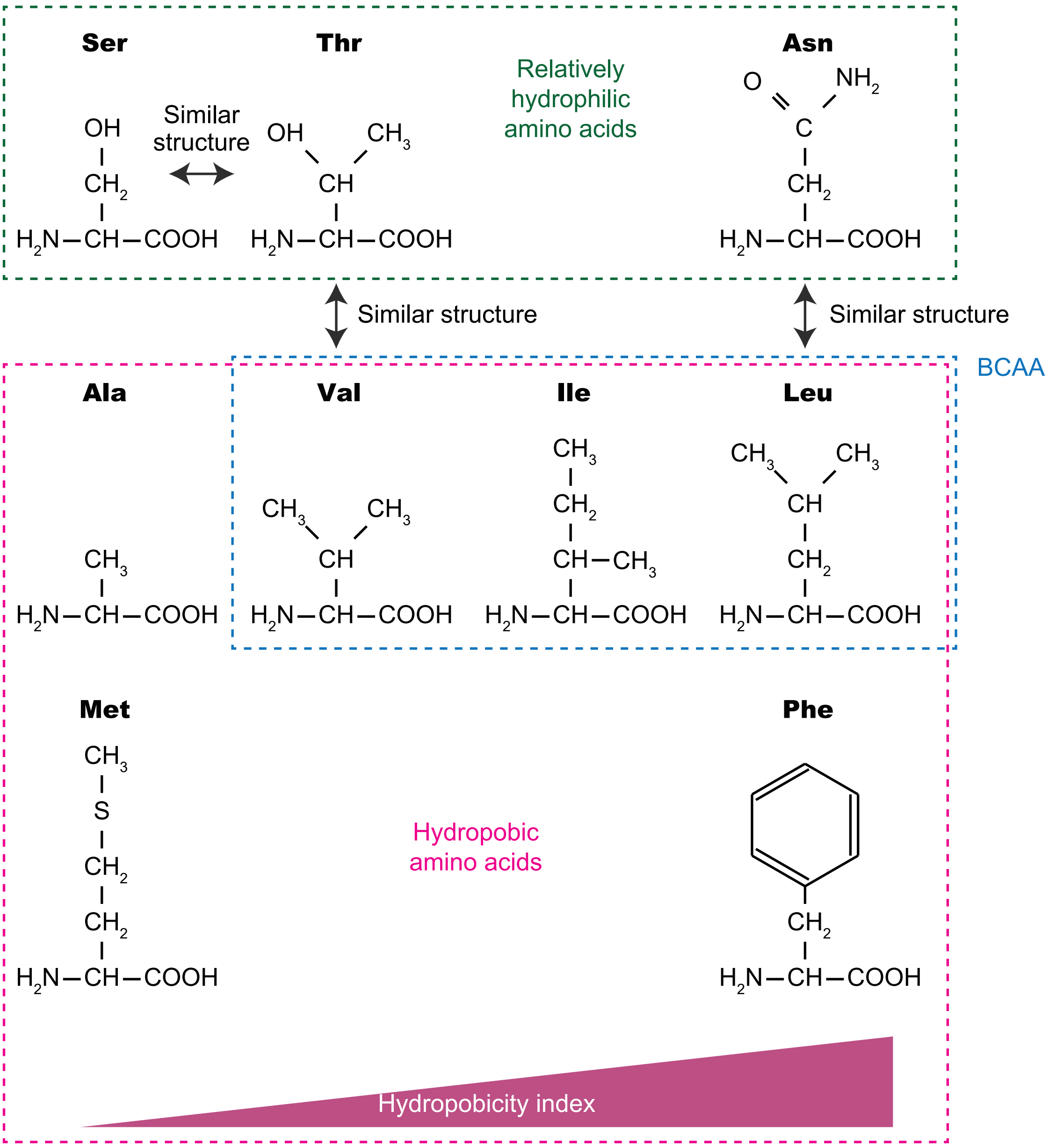

Figure S6. Structure and properties of amino acid side chains used for substitutions.

Schematic representation of side chain structure and properties. The blue-shaded area indicates branched-chain amino acids (BCAA); the magenta-shaded area indicates hydrophobic amino acids. Amino acids are arranged with increasing hydrophobicity index to the right. The green-shaded area indicates hydrophilic amino acids.

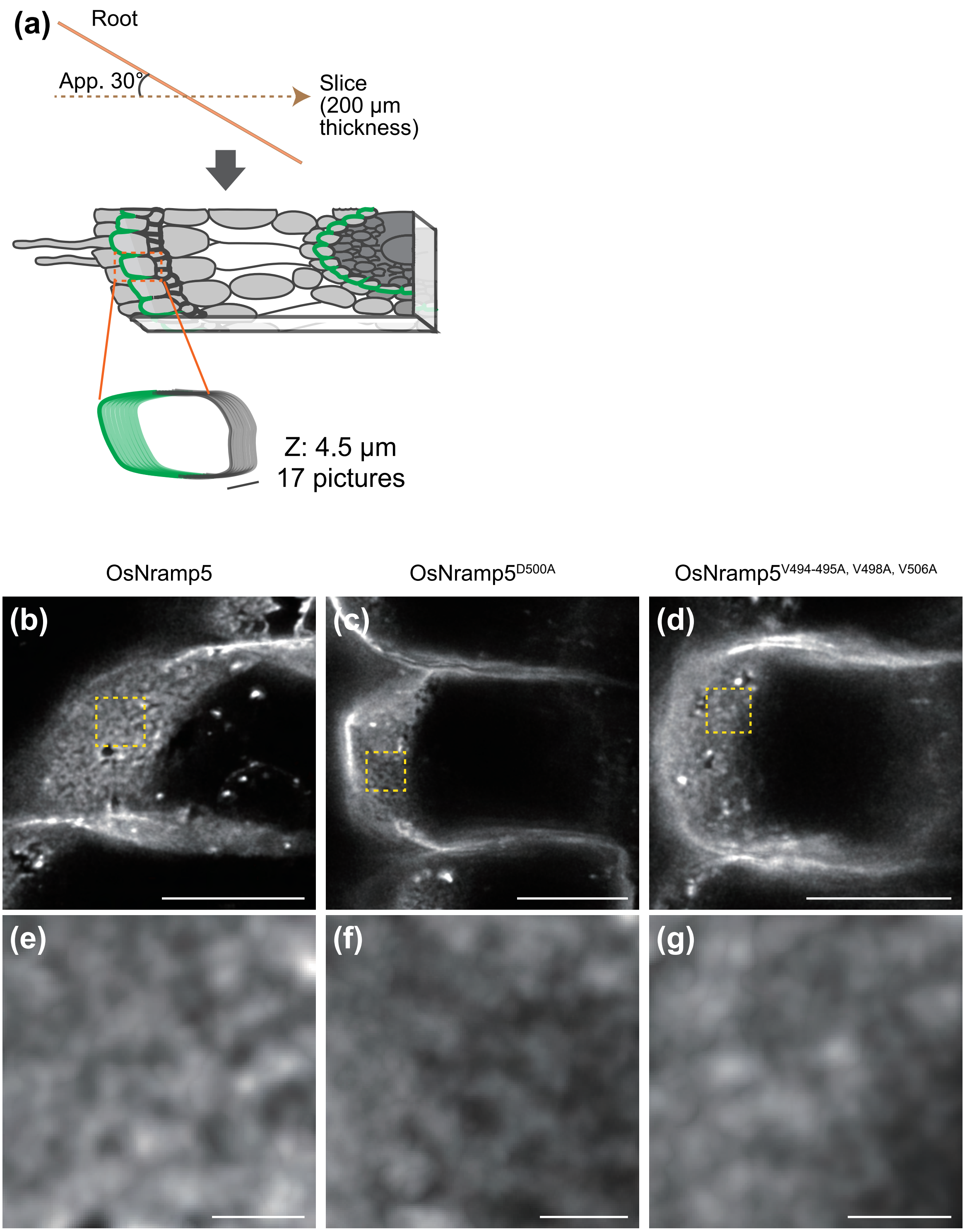

Figure S7. Protein clustering analysis of polarly, weak-polarly, and non-polarly localized OsNramp5 variants in the exodermis.

(a) Schematic of the imaging method for observing protein clustering at the proximal side of exodermis cells. Green indicates predicted protein signal; gray indicates plasma membrane and cell wall. (b-g) Super-resolution confocal images of OsNramp5 (b, e), OsNramp5^{D500A} (c, f), OsNramp5$^{V494-495A,\ V498A,\ V506A}$ (d, g). Yellow-boxed regions in (b-d) are enlarged in (e-g), respectively. Tilted cross-sections (~30°) of mature crown roots from transgenic lines expressing *OsNramp5-Flag* variants were immunostained with anti-Flag antibody. Z-stacks (17 images, total depth 4.5 μm) were merged. Scale bars: 10 μm (b-d) and 1 μm (e-g).

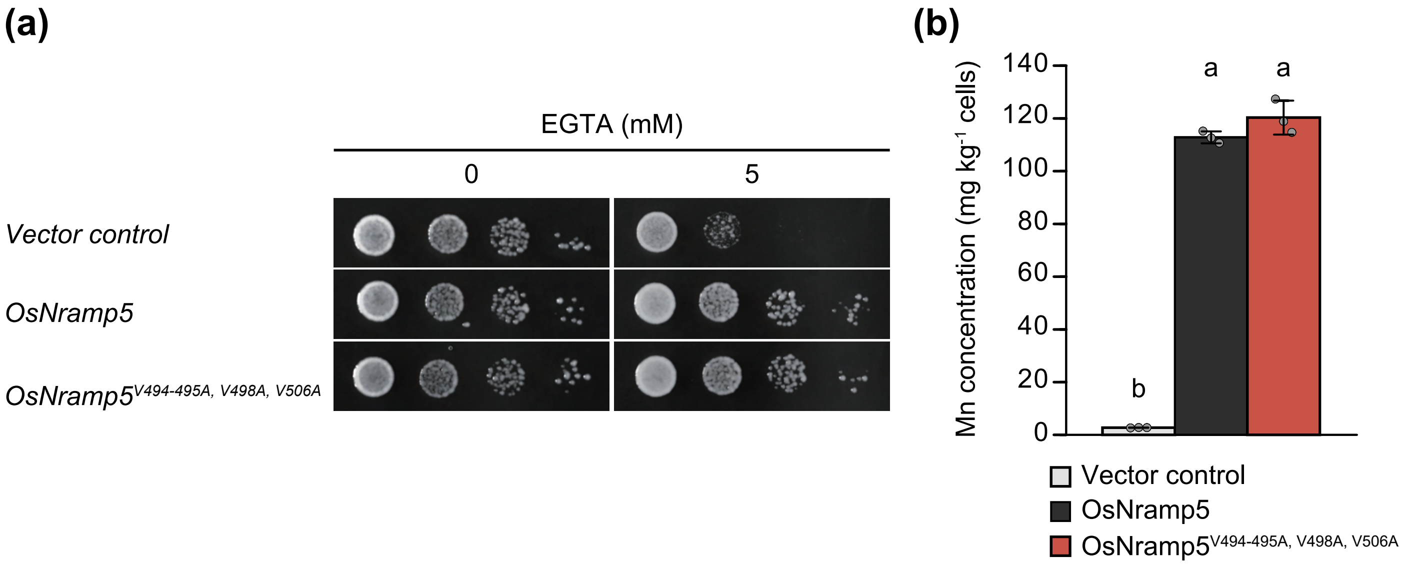

Figure S8. Mn transport activity of OsNramp5 variants in yeast.

(a-b) Growth (a) and Mn accumulation (b) in the Mn uptake-defective yeast strain *Δsmf1* expressing empty vector, *OsNramp5-Flag*, or *OsNramp5*$^{V494\text{-}495A,\ V498A,\ V506A}$*-Flag*. (a) Serial dilutions of yeast were spotted on plates with or without 5 mM EGTA and incubated at 30°C for 2 days. (b) Yeast strains were cultured in liquid SD medium containing 4.6 μM Mn for 5.5 h at 30°C, and cellular Mn concentration was measured by ICP-MS. Data are means ± SD (n = 3). Different letters indicate significant differences ($P < 0.05$, Tukey–Kramer test).

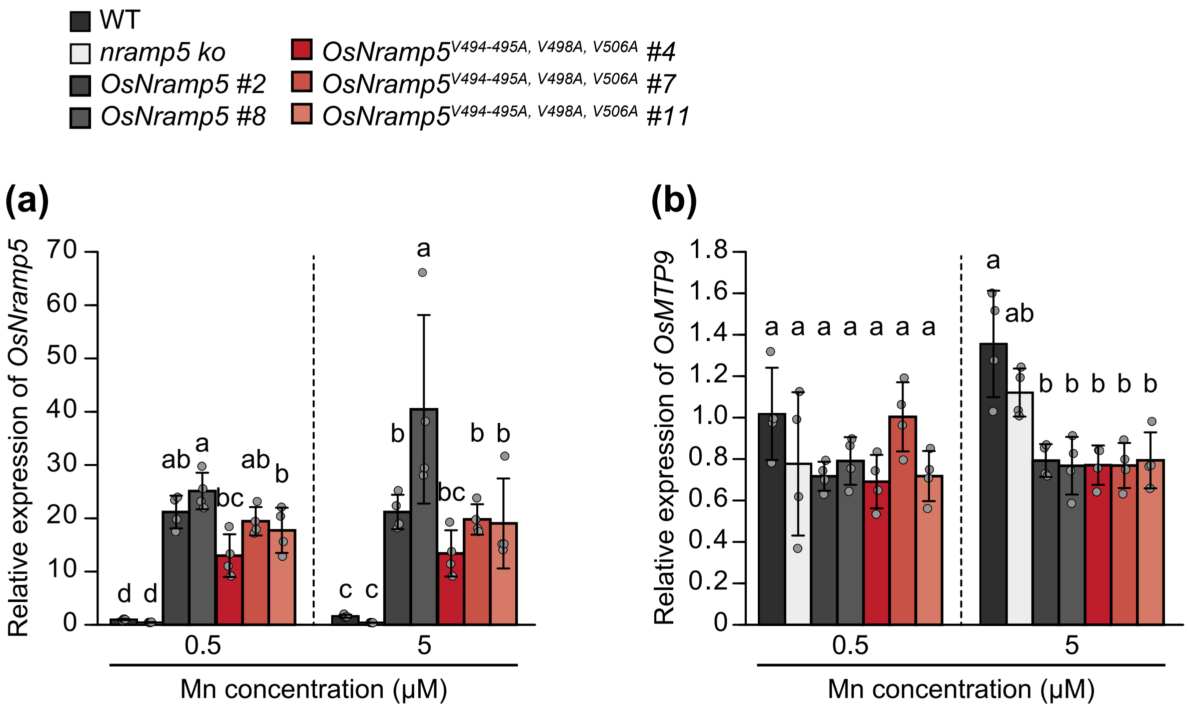

Figure S9. Expression of Mn transporters in roots of transgenic plants expressing polarly or non-polarly localized *OsNramp5*.

(a-b) Relative expression levels of *OsNramp5* (a) and *OsMTP9* (b) in roots of WT, *nramp5* loss-of-function mutant, and transgenic lines expressing *OsNramp5-Flag* or *OsNramp5*$^{V494\text{-}495A,\ V498A,\ V506A}$*-Flag* under 0.5 or 5 µM Mn. Eleven-day-old seedlings pre-grown in 0.5 µM Mn were exposed to 0.5 or 5 µM Mn for 9 days. Expression was normalized to *HistoneH3* and *Ubiquitin*. Data are means ± SD (n = 4). Different letters indicate significant differences ($P < 0.05$, Tukey–Kramer test).

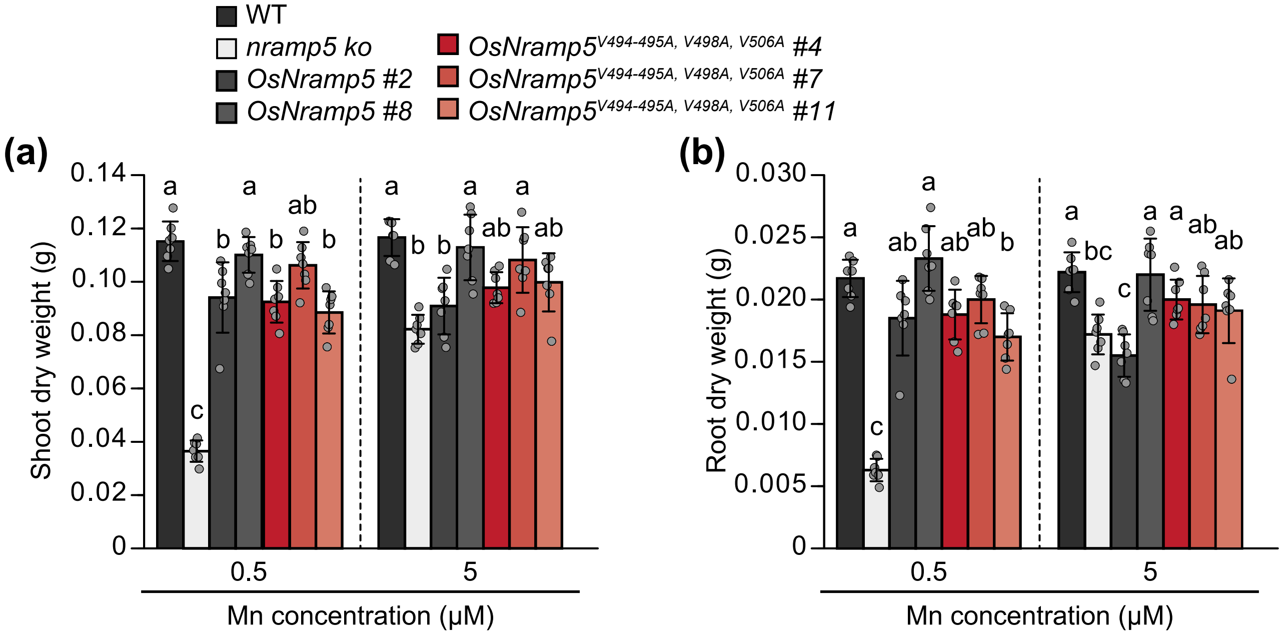

Figure S10. Growth of plants expressing polarly or non-polarly localized *OsNramp5* under 0.5 and 5 µM Mn conditions.

(a-b) Shoot (a) and root (b) dry weight of wild type (WT), *nramp5* loss-of-function mutant, and transgenic lines expressing *OsNramp5-Flag* or *OsNramp5*$^{V494\text{-}495A,\ V498A,\ V506A}$*-Flag* under 0.5 or 5 µM Mn. Eleven-day-old seedlings pre-grown in 0.5 µM Mn were exposed to 0.5 or 5 µM Mn for 9 days. Data are means ± SD (n = 7). Different letters indicate significant differences ($P < 0.05$, Tukey–Kramer test).

```
                  12th TMD                 C-terminal cytosolic region

OsNramp1   478   LTFRKDTV-KFVS--RRELQAGDDTEKAQVATCVADEHSKEPPV--------------   518
OsNramp3   493   LVFRKNRKATLPLLEGDSTVRIVGRDTATEGEGSLGHLPREDISSMQLPQQRTASDLD   550
OsNramp4   494   LTFRKDTVATYVPVPERAQAQVEAGGTPVVDASAADEDQPAPYRKDLADASM------   545
OsNramp5   487   LTIRKDSVVTFVA--DSSLAAVVDAEKADAGDLAVDDDEPLPYRDDLADIPLPR----   538
```

Figure S11. Amino acid sequence alignment of the C-terminal region of polar and non-polar OsNramp members.

Multiple sequence alignment of OsNramp1, OsNramp3, OsNramp4, and OsNramp5 C-termini. Magenta letters indicate Val and Asp residues required for polar localization of OsNramp5; the green letter indicates the Asp residue required for ER export. Blue boxes indicate C-terminal cytosolic regions; brown boxes indicate transmembrane domains (TMD).

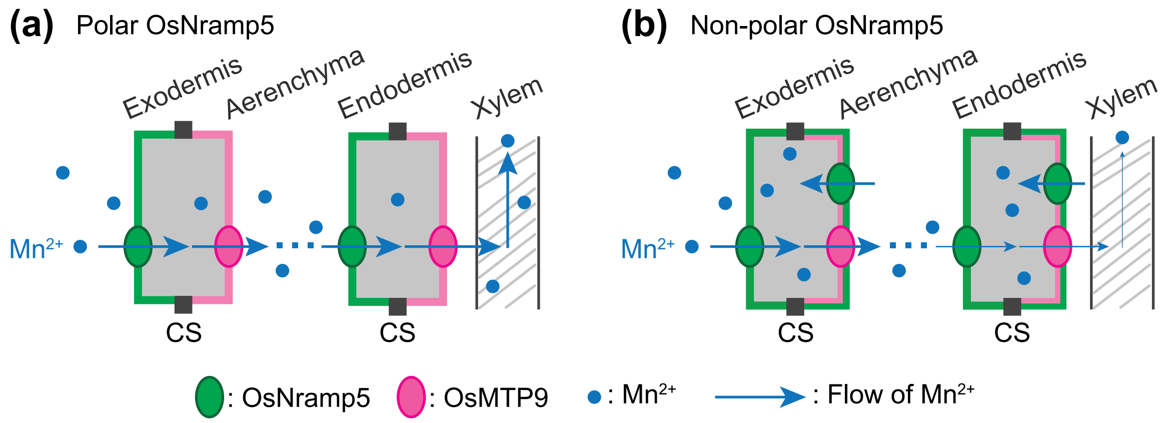

Figure S12. Schematic model of the role of OsNramp5 polar localization in Mn uptake in rice.

(a-b) Proposed function of OsNramp5 polarity in Mn uptake. (a) When OsNramp5 is polarly localized at the distal side of the exodermis and endodermis, it forms a directional transport pathway with the efflux transporter OsMTP9, facilitating efficient Mn uptake. (b) Loss of OsNramp5 polarity results in re-uptake of Mn effluxed by OsMTP9 into the inner apoplastic space, disrupting directional transport and reducing uptake efficiency. Green indicates OsNramp5; magenta indicates OsMTP9. Blue dots represent Mn^{2+}; blue arrows indicate Mn flux. CS, Casparian strip.
